# Supplementary material for: Conditional repair by locally switching the thermal healing capability of dynamic covalent polymers with light
Source: Nat Commun. 2016 Dec 12;7:13623. doi: 10.1038/ncomms13623 (PMC5159900; doi:10.1038/ncomms13623)
Supplement: Supplementary Information — Supplementary Figures 1-23, Supplementary Tables 1-5, Supplementary Notes 1-6, Supplementary Methods, and Supplementary References [file ncomms13623-s1.pdf]

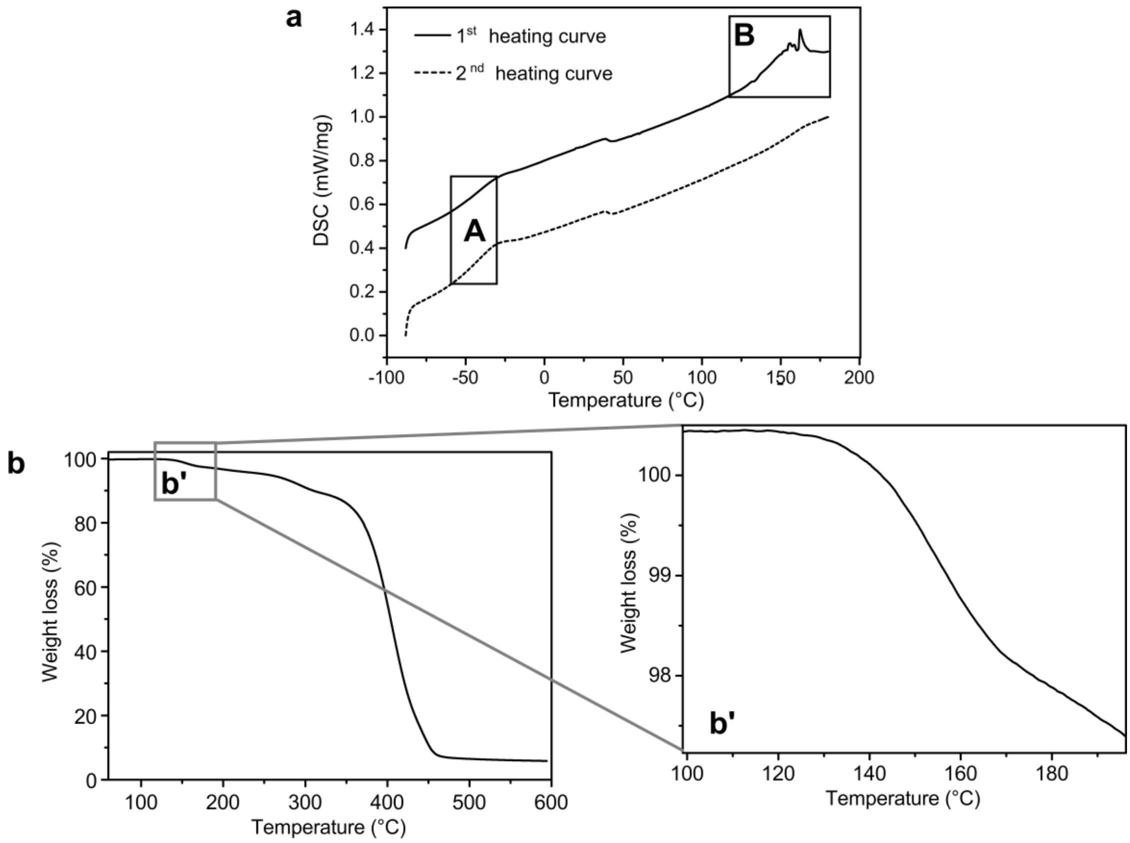

Supplementary Figure 1 | (a-b) DSC and TGA of fpP → P. (**a**) DSC measurements of **fpP** revealing a strong linear decrease from -54 to -34 °C with a glass transition temperature of approximately -44 °C in the first as well as the second heating curve (**A**) and a strong endothermic peak at 120 to 180 °C (retro Diels-Alder reaction and evaporation of furan) (**B**) in the first heating curve. In the second heating curve, another endothermic peak cannot be observed. (**b**) Thermogravimetric analysis (TGA) of **fpP** showing a mass loss of the furan protection group in **b'** in a temperature range of 120 to 170 °C of 2% (theoretical mass loss: 3%). Two other degradation steps follow > 250 °C. For further characterization of **P** see Supplementary Methods and Supplementary Fig. 2, 5, 9, 14, 22, 23.

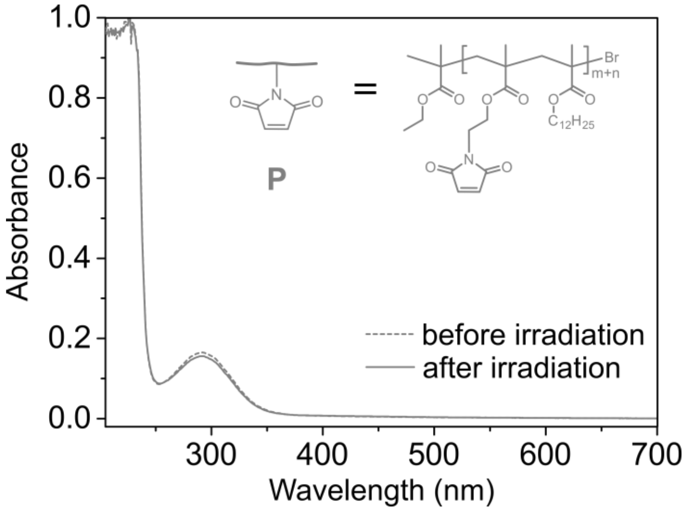

Supplementary Figure 2 | Solid state UV/vis spectroscopy of blank linear P. Stacked normalized solid state UV/vis spectra of **P** before (dashed line) and after illumination with light of 365 nm of a LED for 30 min (solid line) showing no absorption changes, confirming the results shown in Supplementary Fig. 9b. Importantly, optical transparency is provided above ca. 350 nm. For further characterization of **P** see Supplementary Methods and Supplementary Fig. 1, 5, 9, 14, 22, 23.

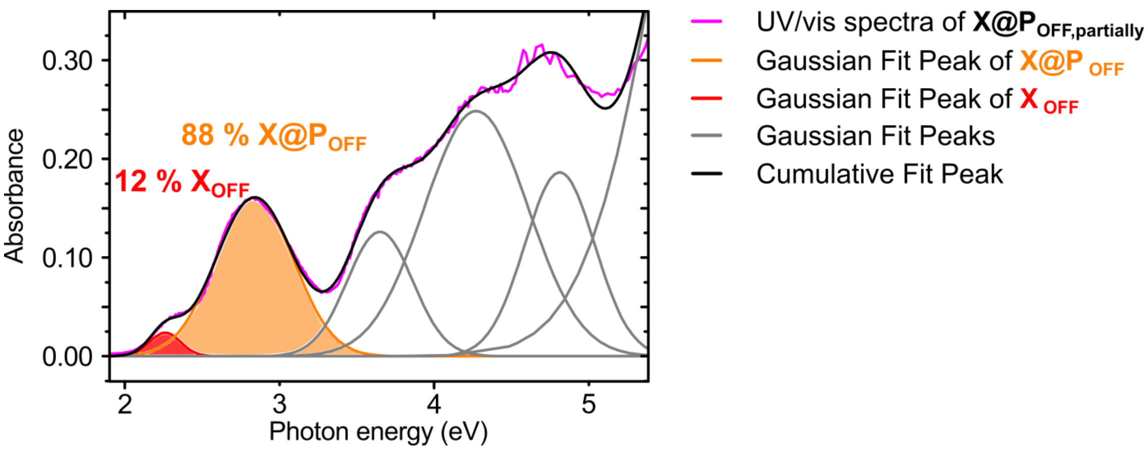

Supplementary Figure 3 | Determination of the conversion of the Diels-Alder crosslinking reaction of X@P$_{\mathbf{OFF}}$ in the photostationary state via UV/vis spectroscopic analysis. UV/vis spectra of **X@P$_{\mathbf{OFF,partially}}$** (magenta line) and the respective Gaussian fits of **X$_{\mathbf{OFF}}$** (red line with filled area) and **X@P$_{\mathbf{OFF}}$** (yellow line with filled area) to estimate their ratio of molar concentrations to be approximately 88% of reacted furan vs. 12% free furan moieties, originating from unreacted crosslinker. For a detailed procedure please see Supplementary Note 1.

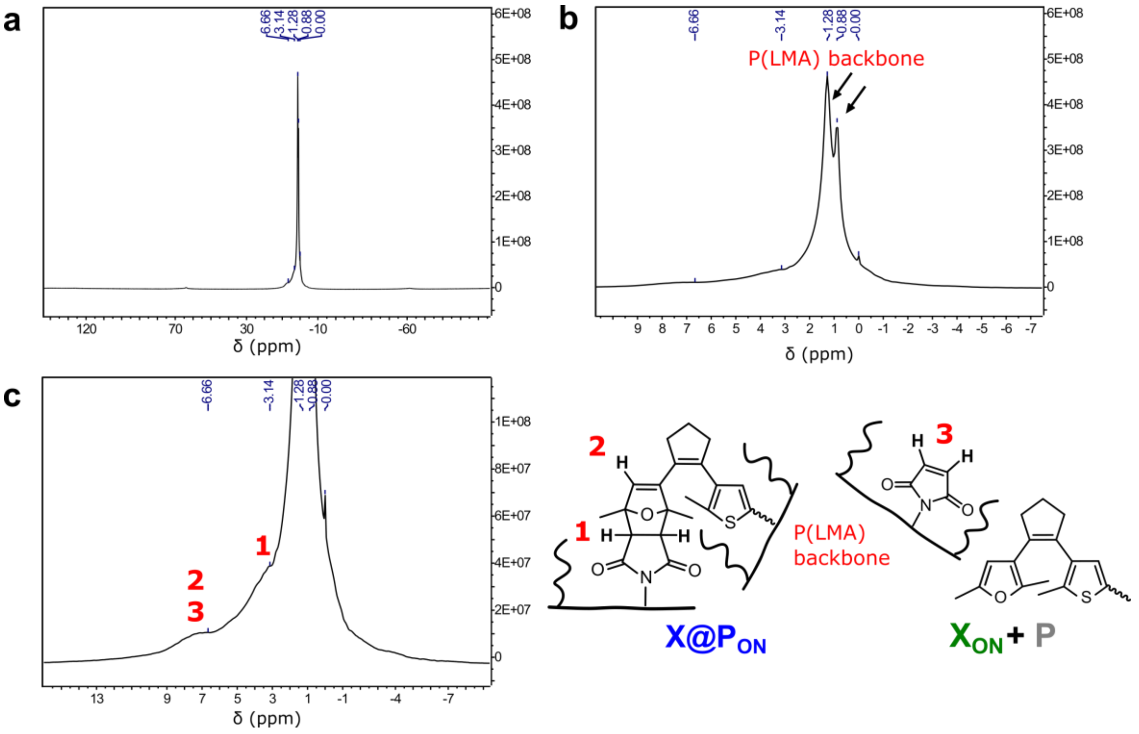

Supplementary Figure 4 | Solid state ^{1}**H-NMR spectroscopy of unlocked, non-illuminated polymer network** $\mathbf{X@P_{ON}}$**.** (**a-c**) ^{1}H MAS NMR spectrum with two zoom-in of $\mathbf{X@P_{ON}}$**.** For polymer network preparation, copolymer **P** (13.2 mol% maleimide content, M_n = 9200 g·mol^{-1}, $Đ$ = 1.26) was mixed with $\mathbf{X_{ON}}$ carrying four furyl-groups in a ratio to assure 0.7 equiv. of furan per maleimide unit. (**a**) Complete ^{1}H MAS NMR spectrum of $\mathbf{X@P_{ON}}$. (**b**) Zoom-in highlighting the peaks related to the poly(lauryl methacrylate) backbone. (**c**) Further zoom-in to peaks belonging to protons of Diels-Alder adduct type crosslinking motifs. For closer examination please see Supplementary Note 2.

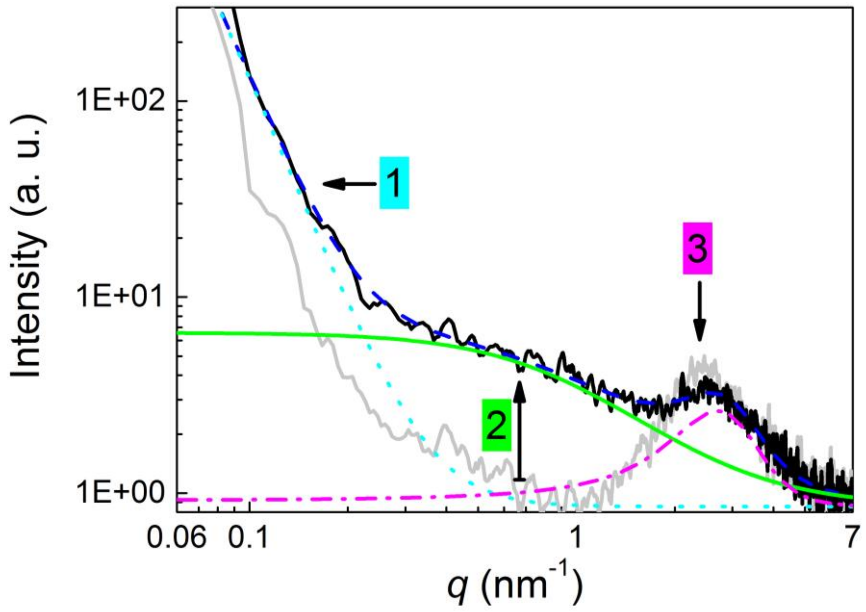

Supplementary Figure 5 | Scattering curves of the copolymer P without crosslinker X_{ON} (gray solid line) and as part of polymer network X@P_{ON} (black solid line) measured at 21 °C. A curve fit for interpretation of the scattering curve of **X@P_{ON}** using $I(q)$ is given exemplarily (blue dashed line, see Supplementary Equation 2). The cyan dashed line represents the scattering contribution $I_1(q)$ from large inhomogeneities with sizes ≥ 52 nm, the green solid curve is the scattering contribution $I_2(q)$ from the polymeric mesh, and the magenta dash-dotted curve represents the scattering contribution $I_3(q)$ from the broad peak. For further information on mesh size of **X@P_{ON}** see Supplementary Table 2 and for detailed explanation see Supplementary Note 3.

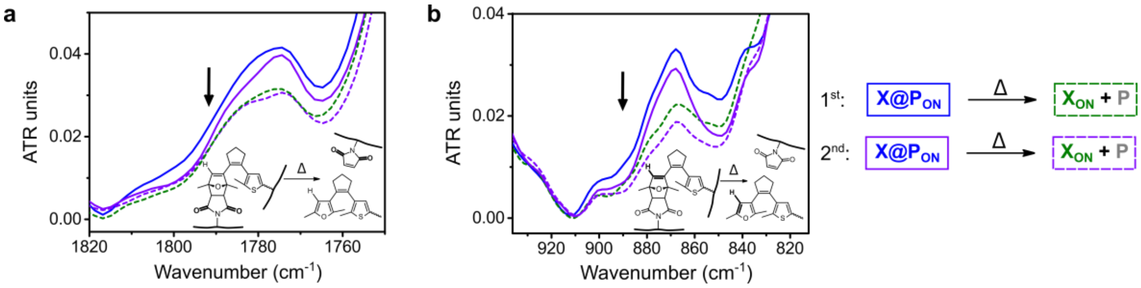

Supplementary Figure 6 | Thermoreversibility of non-illuminated polymer network X@P$_{ON}$. To show the thermoreversibility of the polymer network, FT-IR spectra of **X@P$_{ON}$** (based on 0.7 equiv. of furyl groups per maleimide unit) were recorded before (blue solid line) and after heating to 124 °C for 5 min in vacuum (green dashed line), including a repetition of the heating process after re-crosslinking the material at r.t. for 16 h (purple solid line to purple dashed line). FT-IR spectra are normalized with respect to the band at 2924 cm^{-1} (ν (-CH$_3$)). After heating the samples were immediately cooled with liquid nitrogen to freeze the Diels-Alder equilibrium. (**a-b**) Zoom-in in FT-IR spectra of **X@P$_{ON}$**. (**a**) Focus on the symmetrical C=O stretching mode of succinimide[1–3] at 1774 cm^{-1} and (**b**) the out of plane C-H bending mode at 868 cm^{-1} during two cycles of crosslinking and de-crosslinking (first cycle: blue and green lines, second cycle: purple lines). Note that thermoreversibility of **X@P$_{ON}$** is also confirmed by DSC as well as rheology measurements (Supplementary Fig. 7-8).

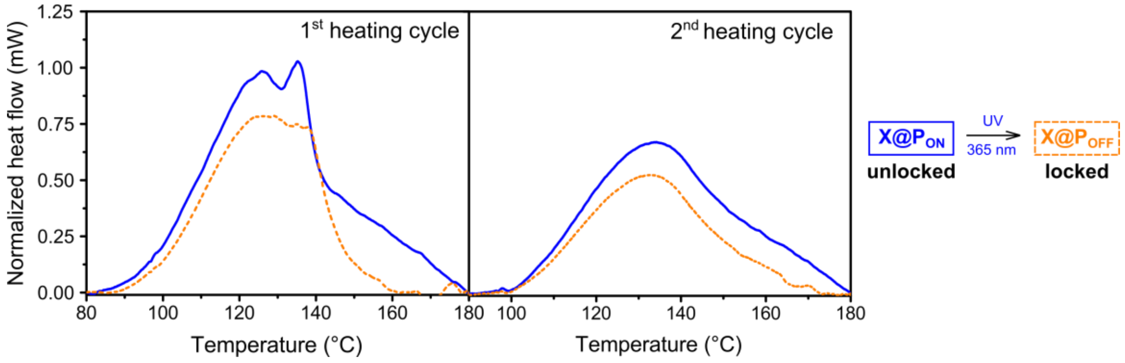

Supplementary Figure 7 | Analysis of endotherms (de-crosslinking of X@P$_{ON}$ and X@P$_{OFF}$) in a temperature range of 80 °C to 180 °C in DSC measurements. Recording of the first and second heating curves was done with a heating rate of 20 K·min^{-1}. For enhanced sensitivity during measurements, a short-chain copolymer **P** (M_n = 4000 g·mol^{-1}, $Đ$ = 1.18) with a higher maleimide content (14 mol%) was mixed with **X$_{ON}$** carrying four furyl-groups in a ratio to assure 1 equiv. of furan per maleimide unit for polymer network preparation. Importantly, the reproducibility of the endotherms in the second heating curve confirms thermoreversibility of the polymer network. Further analysis is carried out in Supplementary Note 5. Note that thermoreversibility of **X@P$_{ON}$** is also confirmed by FT-IR as well as rheology measurements (Supplementary Fig. 6, 8).

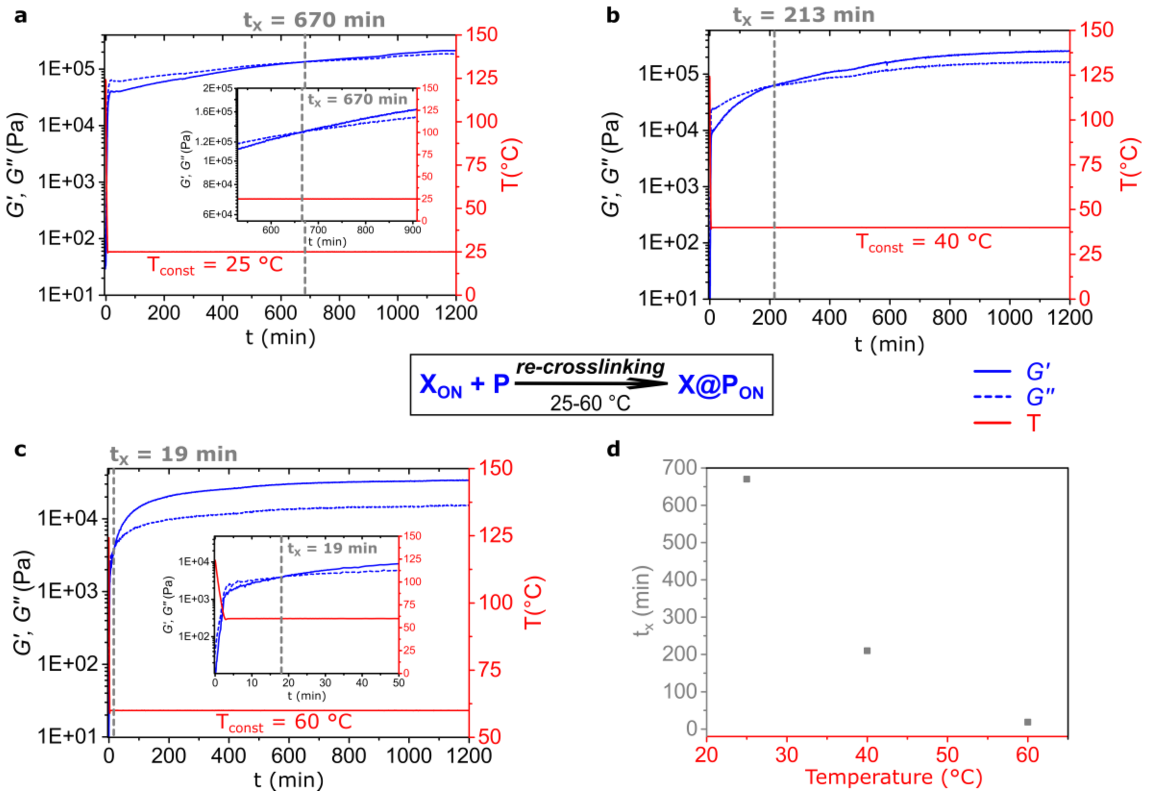

Supplementary Figure 8 | Temperature-controlled sol-gel transition in X@P_{ON}. Rheological monitoring the thermally reversible re-crosslinking of X_{ON} + P → X@P_{ON} , *i.e.* the Diels-Alder reaction, at constant temperatures (25 °C, 40 °C and 60 °C) over time. Note that previous de-crosslinking until an equilibrium of *G'* and *G''* with $G'' > G'$was performed at 124 °C for 30 min. Heating rate to reach constant re-crosslinking temperature: approx. 0.5 $K{\cdot}s^{-1}$. (**a**) Evolution of *G'* and *G''* during re-crosslinking at 25 °C. The crossover point, where $G' = G''$, is reached after t_x = 670 min and can be attributed to a changing of the viscoelastic properties from a liquid to a solid, *i.e.* a recovery of the network properties beyond the crossover point[4]. Noteworthy, both moduli already increase strongly within the first few minutes, due to the fast beginning of the network formation. (**b**) At 40 °C the crossover point is already reached after 213 min and (**c**) at 60 °C after 19 min. The correlation between temperature T and crossover time t_x is represented in (**d**) confirming the dynamics of the Diels-Alder and retro Diels-Alder reaction, *i.e.* crosslinking and de-crosslinking[5,6]. Note that rheology measurements depicted in (**a-c**) furthermore confirm a thermoreversibility of the polymeric network due to a usage of the same sample. Full recovery of crosslinking points at 25 °C and 40 °C can be observed.

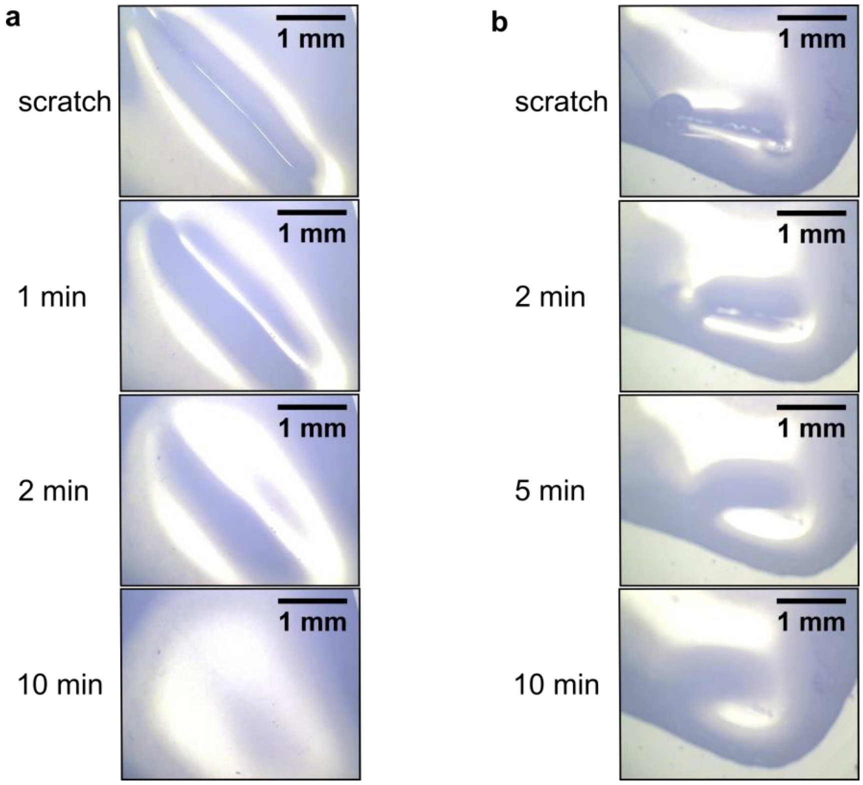

Supplementary Figure 9| (a-b) Scratching tests of the bulk material of blank sample P without crosslinker. For this purpose, a bulk film of poly(LMA-*co*-fpMIMA) (**fpP**) was heated at 130 °C for 3 h to cleave the furan protection group to form poly(LMA-*co*-MIMA) (**P**), followed by tempering at 40 °C for additional 16 h. Afterwards, a model scratch was done with a scalpel in a controlled manner in the mm-scale. Disappearance of the scratch at r.t. was followed by optical microscopy at different time intervals. (**a**) Photographs showing the disappearance of a model scratch at r.t. after 10 min as well as (**b**) the mending of a model scratch after exposing the scratched material to light of 365 nm of a LED for 30 min (20 mA, 4.2 V) at r.t. Both experiments demonstrate that the low T_g and flow of the material are conserved and therefore not hindered by photochemical[7,8] or thermal crosslinking[9] of the linear polymer **P** - leading to a disappearance of the scratches. Mending was followed with an optical microscope. For further characterization of **P** see Supplementary Methods and Supplementary Fig. 1, 2, 5, 14, 22, 23.

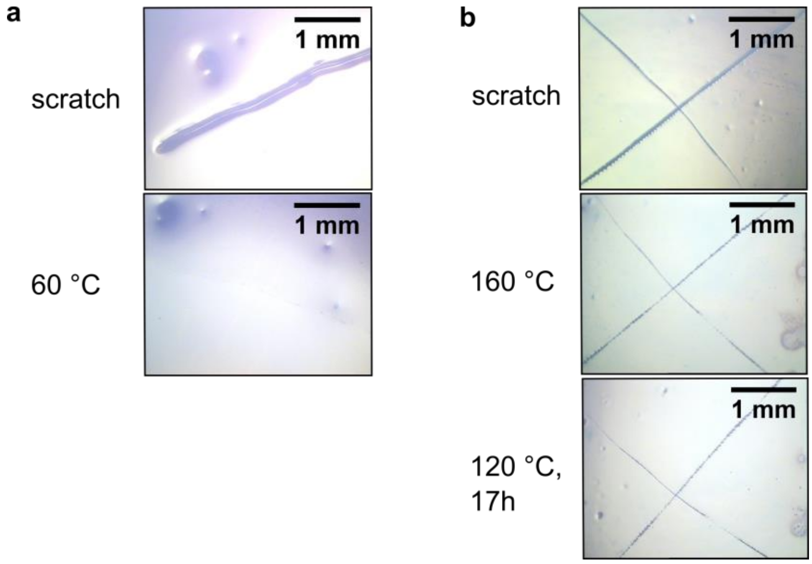

Supplementary Figure 10 | (a-b) Scratching tests of X@P$_{ON}$ (without light control) upon variation of crosslinking density. For preparation of a film of **X@P$_{ON}$**, furyl-protected copolymer **fpP** and crosslinker **X$_{ON}$** were dissolved in a minimum amount of dichloromethane and drop-casted onto a glass slide. The mixture was heated at 130 °C for 3 h, followed by annealing at 40 °C for 16 h. Then, the surface of the polymer network **X@P$_{ON}$** was scratched in a controlled manner. Healing of the scratch at different temperatures was followed by optical microscopy. (**a**) Low crosslinking density: Photographs showing the mending of a model scratch in **X@P$_{ON}$** at 60 °C (network preparation by mixing **P** (6 mol% maleimide content, M_n = 4000 g·mol^{-1}, $Đ$ = 1.13) and **X$_{ON}$** carrying four furyl-groups in a ratio to assure 1 equiv. of furyl groups per maleimide unit). (**b**) Higher crosslinking density: Photographs showing non-healable scratches in **X@P$_{ON}$** at 120 °C and 160 °C (network preparation by mixing **P** (12 mol% maleimide content, M_n = 5000 g·mol^{-1}, $Đ$ = 1.08) and **X$_{ON}$** carrying four furyl-groups in a ratio to assure 1 equiv. of furyl groups per maleimide unit).

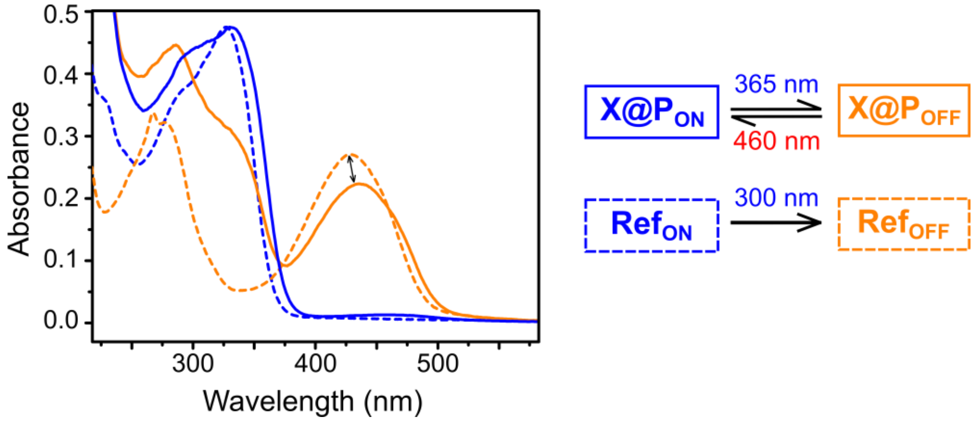

Supplementary Figure 11 | UV/vis spectra for determination of the photoconversion of X@P$_{OFF}$ in the photostationary state. UV/vis spectra of **X@P$_{ON}$** and **X@P$_{OFF}$** (solid lines) in comparison to a small molecule reference compound (dashed lines) in its 100% ring-open state (**Ref$_{ON}$**) and 100% ring-closed state (**Ref$_{OFF}$**). The corresponding determination of the photoconversion is explained in Supplementary Note 4. For detailed structural information on **Ref$_{ON}$** and **Ref$_{OFF}$**, see Supplementary Fig. 16.

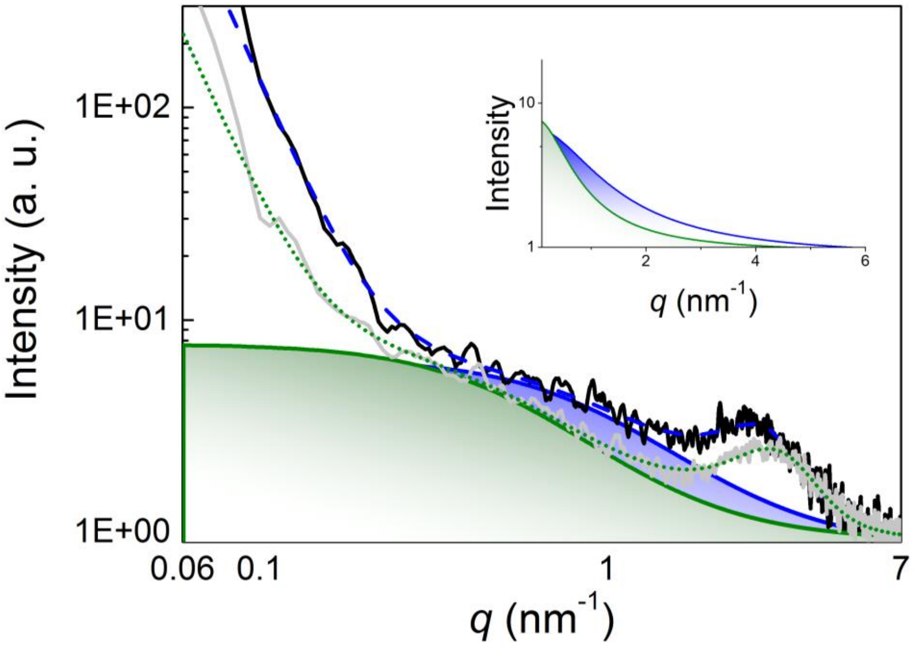

Supplementary Figure 12 | SAXS curves of polymer network X@P$_{ON}$ measured at 21 °C (black solid curve) and after 30 min at 120 °C (gray solid curve) to form X$_{ON}$ + P. Curve fits using $I(q)$ are given as blue dashed and green dotted curve, respectively. The scattering contributions from the network $I_2(q)$ at 21 °C and 120 °C are highlighted as filled area under the curves (blue and green, respectively). The inset shows a log-linear plot of the $I_2(q)$ curves at 21 °C (blue) and 120 °C (green). For further information on mesh sizes see Supplementary Table 2 and for detailed explanation see Supplementary Note 3.

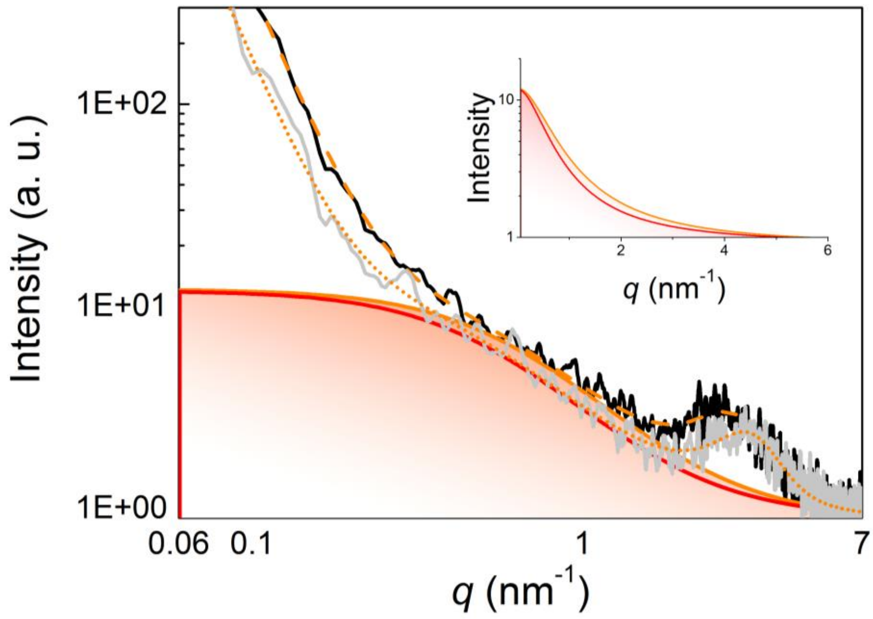

Supplementary Figure 13 | SAXS curves of the locked polymer network X@P$_{OFF}$ measured at 21 °C (black solid curve) and after 30 min at 120 °C (gray solid curve). Curve fits using $I(q)$ are given as yellow dashed and yellow dotted curve, respectively. The scattering contributions from the network $I_2(q)$ at 21 °C and 120 °C are highlighted as filled area under the curves (yellow and red, respectively). The inset shows a log-linear plot of the $I_2(q)$ curves at 21 °C (yellow) and 120 °C (red). For further information on mesh sizes see Supplementary Table 3 and for detailed explanation see Supplementary Note 3.

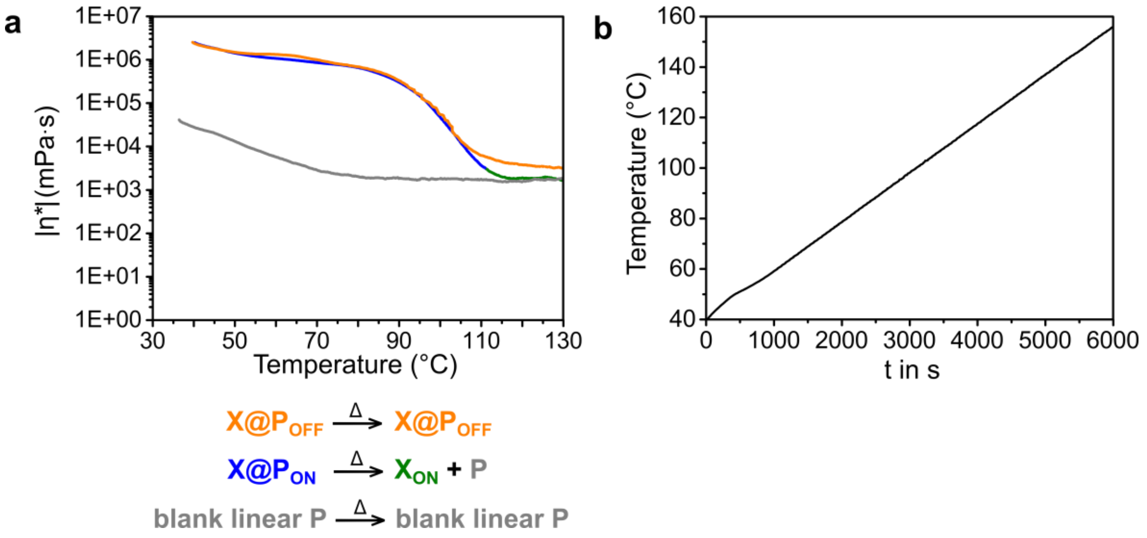

Supplementary Figure 14 | Mechanical properties of linear polymer P and polymer networks. (**a**) Temperature-dependence of the complex viscosity $|\eta|^*$ of blank sample **P** (gray line) and polymer networks $\mathbf{X@P_{ON}}$ (blue → green line) and $\mathbf{X@P_{OFF}}$ (yellow line) (heating at 0.02 $K{\cdot}s^{-1}$). Valid in a temperature range of **40 °C to 105 °C**: $|\eta|^*$ ($\mathbf{X@P_{ON}}$) ≈ $|\eta|^*$ ($\mathbf{X@P_{OFF}}$) ≫ $|\eta|^*$ (**P**). The polymer networks $\mathbf{X@P_{ON}}$ and $\mathbf{X@P_{OFF}}$ in that low temperature range reveal a drastically increased complex viscosity in contrast to the blank, linear polymer **P**. Valid in a temperature range of **117 °C to 130 °C**: $|\eta|^*$ (**P**) ≈ $|\eta|^*$ ($\mathbf{X_{ON}}$ + **P**) ≈ 2$|\eta|^*$ ($\mathbf{X@P_{OFF}}$). Only the complex viscosity of $\mathbf{X@P_{ON}}$ reaches the mean values of **P** upon heating to temperatures higher than ca. 110 °C. In strong contrast, the complex viscosity of locked network $\mathbf{X@P_{OFF}}$ remains higher than $\mathbf{X@P_{ON}}$ and **P**, thus different material properties occur in a high temperature region confirming the light-induced locking and de-locking of the material's crosslink density. (**b**) Linearity of heating ramp applied for rheology measurements shown here (**a**) and in the main article.

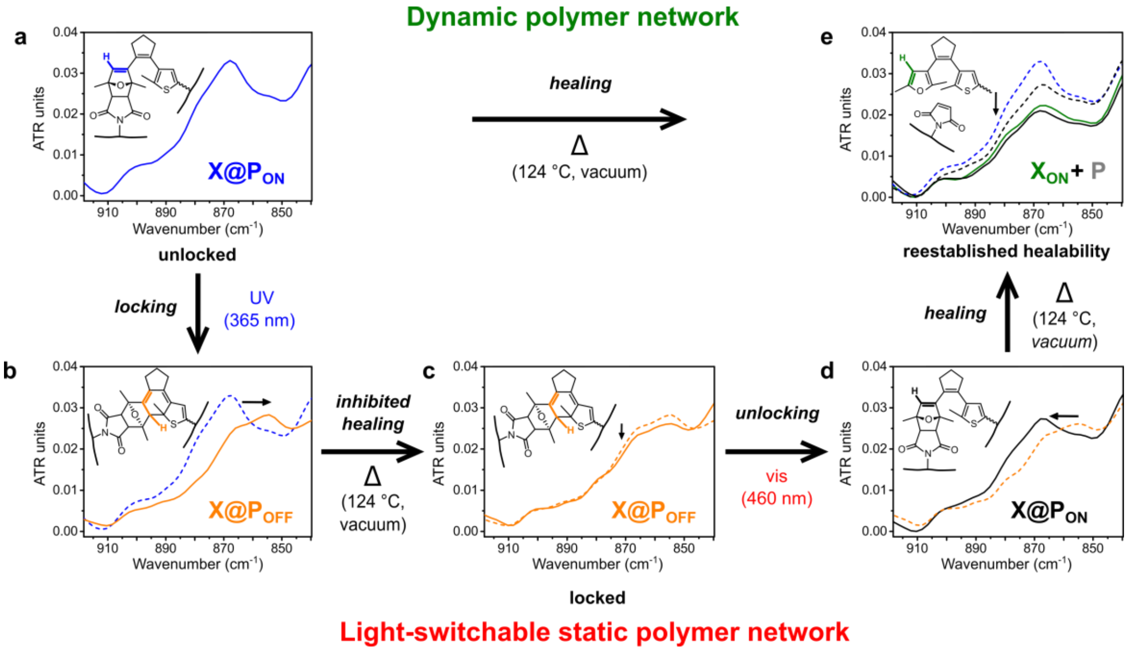

Supplementary Figure 15 | (a-e) FT-IR spectra during a complete light-controlled locking and de-locking cycle of the polymer network focusing on the out of plane C-H bending mode of the vinyl group. (**a**) **X@P$_{ON}$**'s vinyl group at 868 cm^{-1}. (**b**) Shift to 854 cm^{-1} in **X@P$_{OFF}$** (yellow solid line). (**c**) Retaining the vibration mode of **X@P$_{OFF}$** upon heating. (**d**) Back shift to 868 cm^{-1} in regenerated **X@P$_{ON}$** (black solid line). (**e**) Strong decrease of the C-H bending mode of the furyl moiety of the unlocked (blue dashed line) and regenerated **X@P$_{ON}$** (black dashed line) to **X$_{ON}$** and **P** (green and black solid lines). FT-IR spectra of the polymer system are normalized with respect to the band at 2924 cm^{-1} (ν (-CH$_3$)). After heating the samples were immediately cooled with liquid nitrogen to freeze the Diels-Alder equilibrium. For comparison of the C-H bending mode to the small reference compounds **Ref$_{ON}$** and **Ref$_{OFF}$** see Supplementary Fig 16.

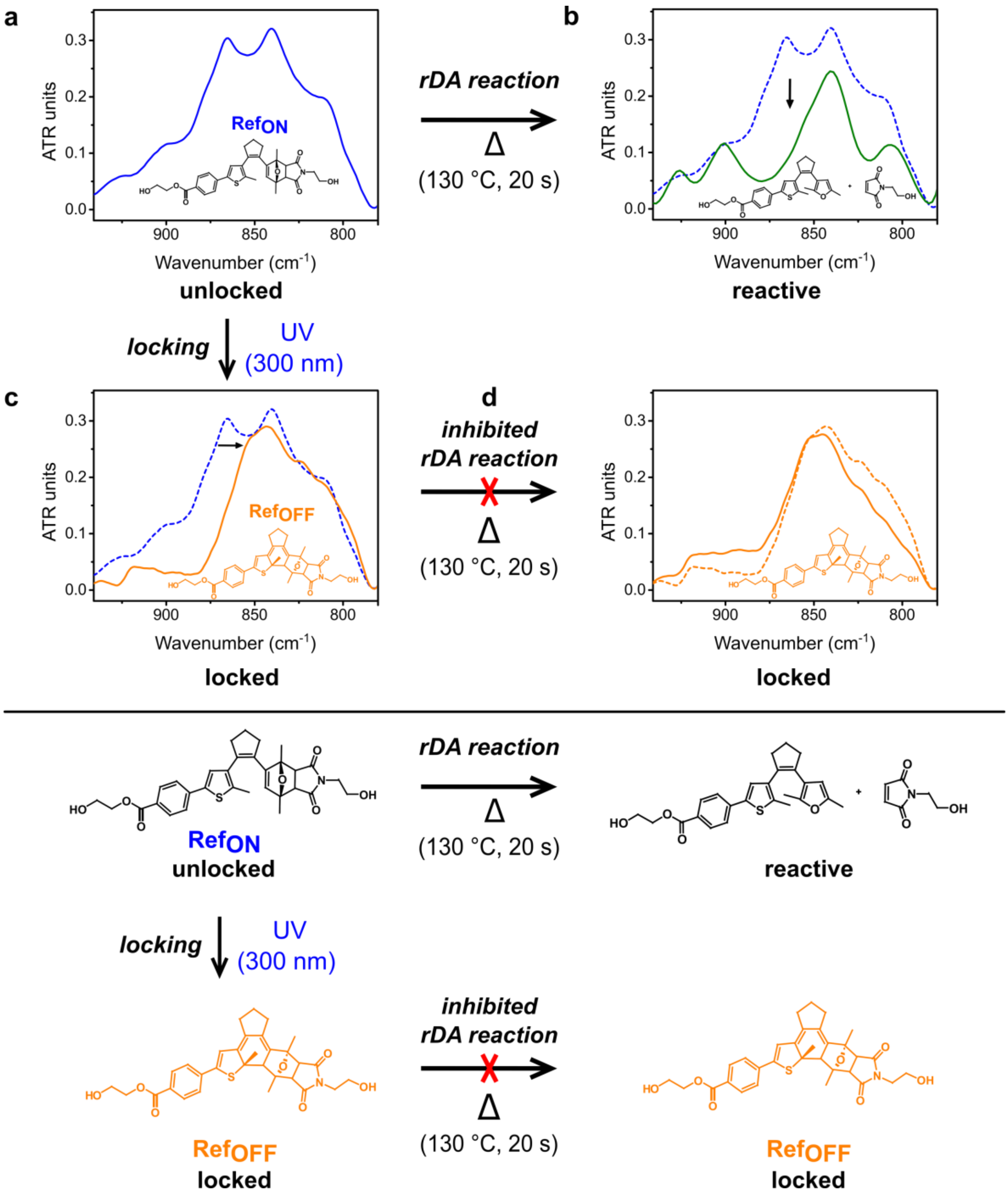

Supplementary Figure 16 | (a-d) FT-IR spectra focusing on the out of plane C-H bending mode of the vinyl group in the small molecule reference compound $\mathbf{Ref_{ON}}$. (**a**) $\mathbf{Ref_{ON}}$'s vinyl group at 866 cm^{-1}. (**b**) Retro Diels-Alder (rDA) reaction of $\mathbf{Ref_{ON}}$ (blue dashed line to green solid line). (**c**) Shift to 852 cm^{-1} in 100% ring-closed $\mathbf{Ref_{OFF}}$ (yellow solid line). (**d**) Inhibited rDA reaction of $\mathbf{Ref_{OFF}}$ upon heating (yellow dashed line to yellow solid line). FT-IR spectra are normalized with respect to the band at 1690 cm^{-1}. After heating the samples were immediately cooled with liquid nitrogen to freeze the Diels-Alder equilibrium.

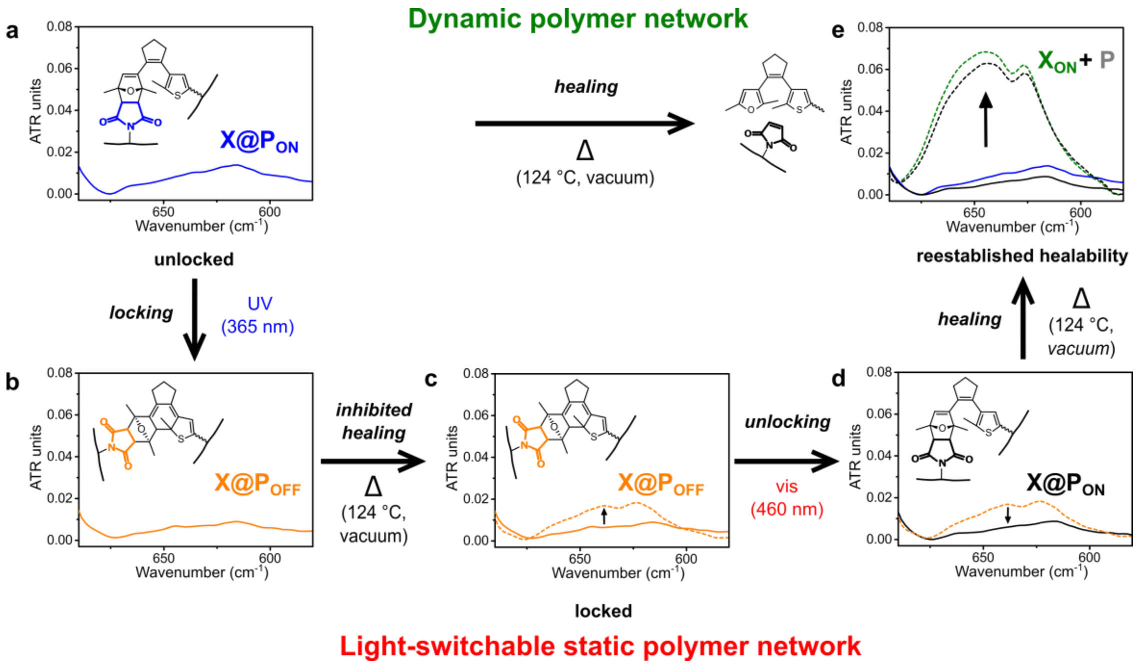

Supplementary Figure 17 | (a-e) FT-IR spectra during a complete light-controlled locking and de-locking cycle of the polymer network focusing on the ring bending mode of maleimide[8,10] during (de-)crosslinking. (**a-b, d**) Suppressed ring bending mode of maleimide in **X@P$_{\mathbf{ON}}$** (blue line) (**a**), **X@P$_{\mathbf{OFF}}$** (yellow line) (**b**) and in regenerated **X@P$_{\mathbf{ON}}$** (black line) (**d**). (**c**) Slight appearance of the ring bending mode of maleimide at 638 cm^{-1} in **X@P$_{\mathbf{OFF}}$** upon heating (yellow dashed line). (**e**) Strong appearance of the ring bending mode of maleimide at 645 cm^{-1} due to de-crosslinking from unlocked (blue solid line) as well as regenerated (black solid line) **X@P$_{\mathbf{ON}}$** to **X$_{\mathbf{ON}}$** and **P** (green and black dashed lines). FT-IR spectra of the polymer system are normalized with respect to the band at 2924 cm^{-1} (ν (-CH_3)). After heating the samples were immediately cooled with liquid nitrogen to freeze the Diels-Alder equilibrium.

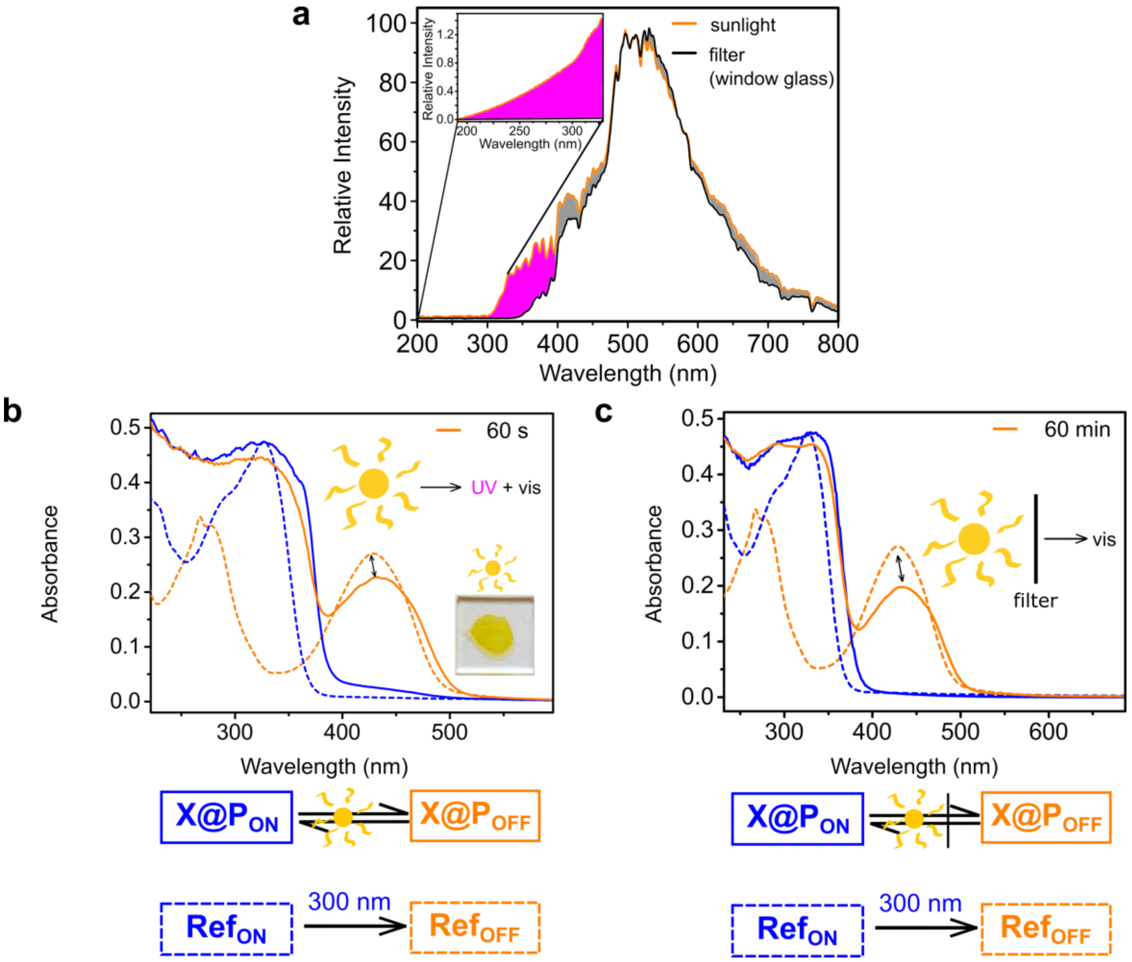

Supplementary Figure 18 | Normalized UV/vis spectra. (**a**) UV/vis spectra of sunlight with and without filter window glass with highest intensities in the area of approx. 500 to 600 nm. The magenta filled area illustrates a higher amount of UV-light without filter. (**b**) Solid state UV/vis spectra showing the composition of the photostationary state for **X@P$_{ON}$** → **X@P$_{OFF}$** under sun light with a conversion to approx. 80% ring-closed isomer (**X@P$_{OFF}$**, yellow line). (**c**) After photochemical **X@P$_{ON}$** → **X@P$_{OFF}$** conversion to 83% ring-closed isomer with light of 365 nm of a LED (compare Supplementary Fig. 11 and Note 4 for photoconversion of **X@P$_{OFF}$** in the photostationary state), **X@P$_{OFF}$** is exposed to ambient light conditions behind a window glass. After 60 min still 73% ring-closed isomer (**X@P$_{OFF}$**) remain, revealing a very slow conversion to **X@P$_{ON}$** over a period of many hours. **X@P$_{OFF}$** is reasonably stable towards ambient light conditions (under sun light as well as behind a typical window) and thus, no handling under special dark conditions is necessary. For more detailed information please see Supplementary Note 6. PSS calculation was performed according to Supplementary Fig. 11 and Supplementary Note 4.

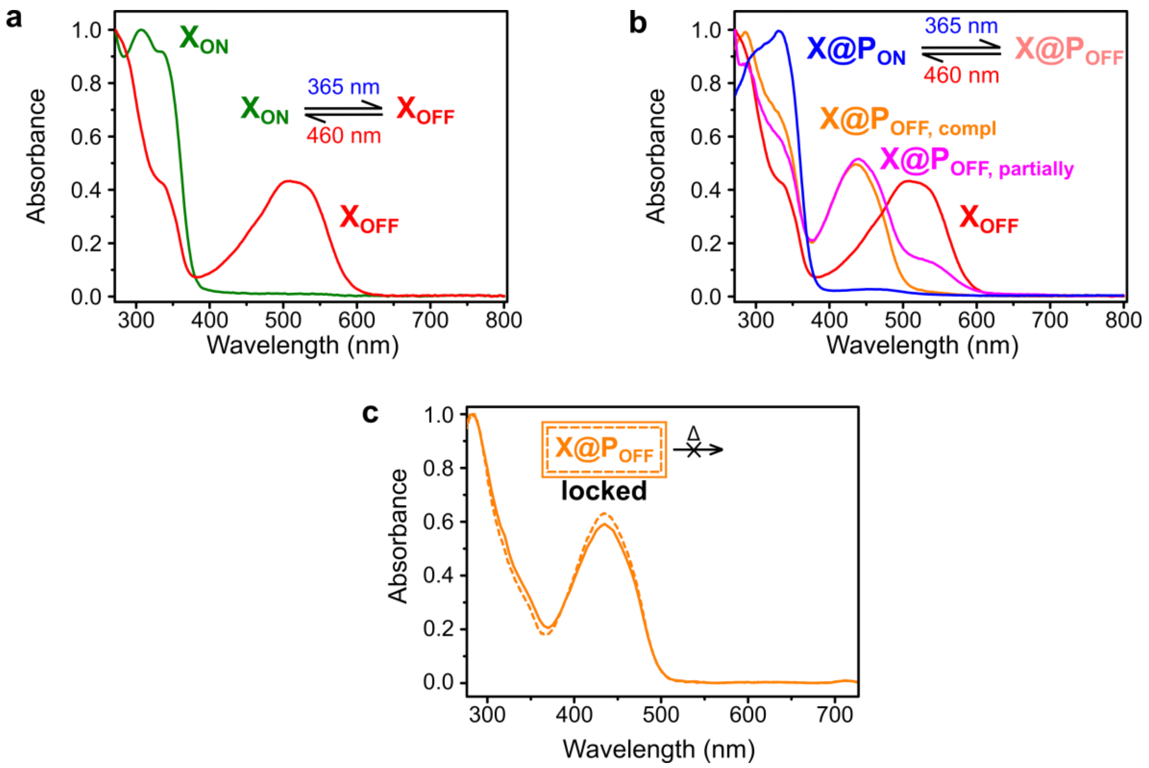

Supplementary Figure 19 | (a-c) Solid state UV/vis spectroscopy of crosslinker and polymer networks. (**a**) Normalized UV/vis spectrum of $\mathbf{X_{ON}}$ in unreactive poly(LMA) upon illumination (green line, 365 nm LED, 3 min) to form ring-closed isomer $\mathbf{X_{OFF}}$ (red line; reddish coloring of polymer film). Reversal was complete by quantitative ring-opening to reform $\mathbf{X_{ON}}$ (460 nm LED, 9 min). (**b**) Normalized UV/vis spectra of $\mathbf{X@P_{ON}}$ (blue line), completely crosslinked $\mathbf{X@P_{OFF,compl}}$ (yellow line, 365 nm, 8 min, 0.6 : 1 furan : maleimide; yellow coloring of polymer film) and partially crosslinked $\mathbf{X@P_{OFF,partially}}$ (magenta line, 365 nm LED, 7 min, 0.7 : 1 furan : maleimide; pinkish coloring of polymer film) in comparison to $\mathbf{X_{OFF}}$ in unreactive poly(LMA) (red line). Complete reversion of $\mathbf{X@P_{OFF,compl\ /\ partially}}$ by quantitative ring-opening to reform $\mathbf{X@P_{ON}}$ was achieved by 460 nm LED light illumination (30 min). (**c**) Normalized UV/vis spectra of $\mathbf{X@P_{OFF}}$ before (dashed line) and after heating (solid line) to 130 °C for 2 min showing no significant changes and thus proving thermal stability of locked $\mathbf{X@P_{OFF}}$. The polymer film was tempered on a quartz plate at 130 °C for 2 min, followed by recording the UV/vis spectrum.

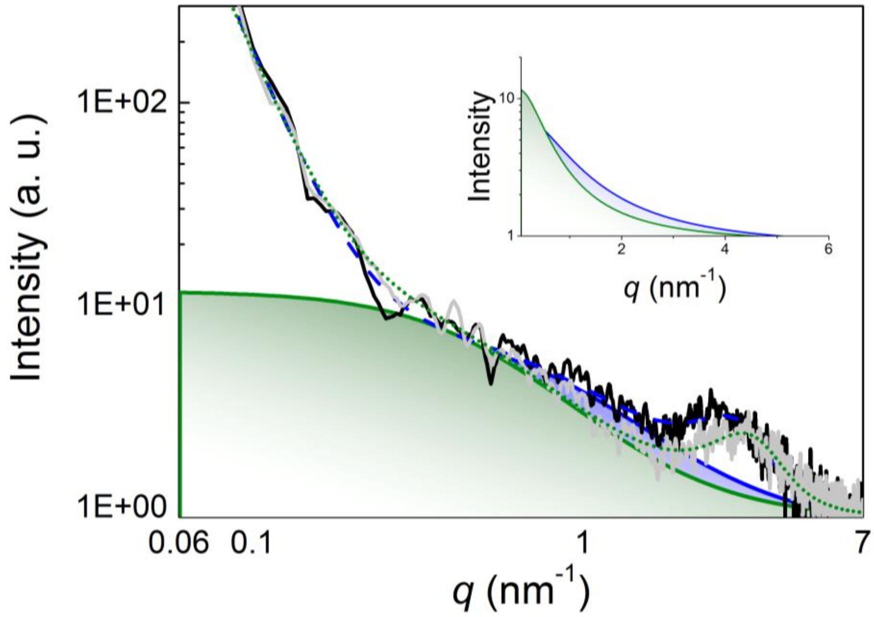

Supplementary Figure 20 | SAXS curves of the regenerated polymer network X@P$_{ON}$ with a reestablished healability measured at 21 °C (black solid curve) and after 30 min at 120 °C (gray solid curve) to form X$_{ON}$ + P. Curve fits using $I(q)$ are given as blue dashed and green dotted curve, respectively. The scattering contributions from the network $I_2(q)$ at 21 °C and 120 °C are highlighted as filled area under the curves (blue and green, respectively). The inset shows a log-linear plot of the $I_2(q)$ curves at 21 °C (blue) and 120 °C (green). For further information on mesh sizes see Supplementary Table 5 and for detailed explanation see Supplementary Note 3.

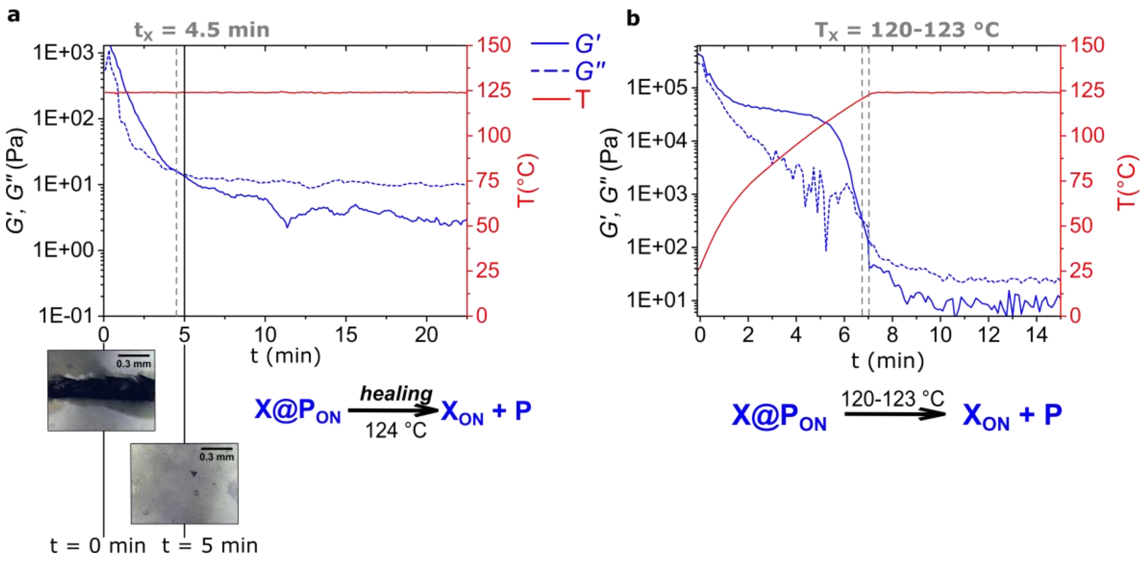

Supplementary Figure 21 | Thermo-rheological behavior of X@P$_{ON}$ at constant healing temperature (a) or with fast heating rate (b). (**a**) Evolution of the time-dependent storage (*G'*) and loss (*G''*) modulus at healing temperature 124 °C which was applied for scratching tests[11]. After t_x = 4.5 min at 124 °C (gray dashed line), the typical crossover with $G' = G''$ from $G' > G''$ to $G' < G''$occurs changing the materials viscoelastic properties from a solid to a liquid, meaning de-crosslinking of the network (**X@P$_{ON}$** → **X$_{ON}$** + **P**) (*i.e.* "inverse gel point"). The equilibration of *G'* and *G''* takes approximately 13 min, indicating that scratch healing (5 min at 124 °C, see Fig. 2 in MS for scratching tests) already takes place before the Diels-Alder equilibrium is established, *i.e.* that not all crosslinks have to be broken to accomplish healing [5]. This is confirmed by further scratching tests in a for rheology measurements representative thicker crosslinked film, where the scratch completely healed after heating at 120-124 °C for 5 min (see micrographs of scratch before and after healing at t = 0 min and t = 5 min). (**b**) Evolution of the time- and temperature dependent storage (*G'*) and loss (*G''*) modulus with a heating rate (13.5 K·min^{-1}) to reach healing temperature of 124 °C as fast as possible. An approximation of *G'* and *G''* occurs between 115 °C and 123 °C with a crossover point at approx. 120-123 °C confirming a healing temperature in that region.

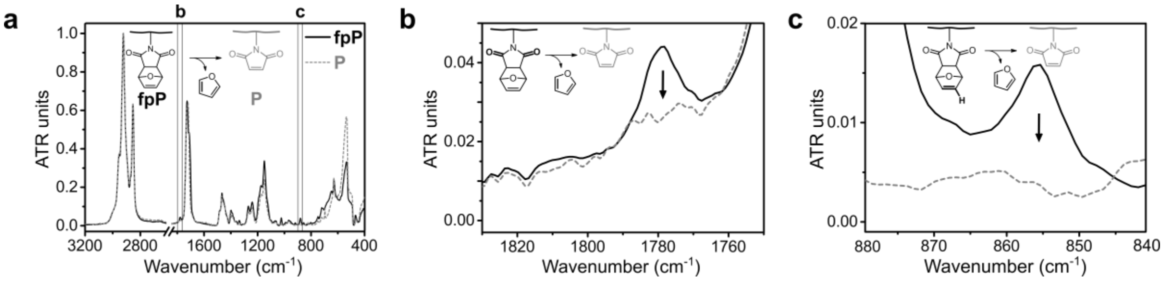

Supplementary Figure 22 | (a-c) FT-IR spectroscopic analysis of the deprotection of furyl-protected, inactive linear polymer fpP to form non-protected, active linear polymer P via cleavage of the furan protection group. (**a**) Complete FT-IR spectra of **fpP** (before heating, black solid line) and of **P** (after heating of **fpP** in the bulk to 130 °C in a drying oven for 1 h, gray dashed line). Spectra are normalized with respect to the band at 2923 cm^{-1}. (**b**) Zoom-in of the C=O stretching mode of the succinimide moiety in the Diels-Alder adduct[2,3,12] in **fpP** at 1778 cm^{-1} as well as **c**, the out of plane CH-bending mode of the vinylene group of the furyl moiety[13,14] at 855 cm^{-1}, both not visible in **P**. For further characterization of **P** see Supplementary Methods and Supplementary Fig. 1, 2, 5, 9, 14, 23.

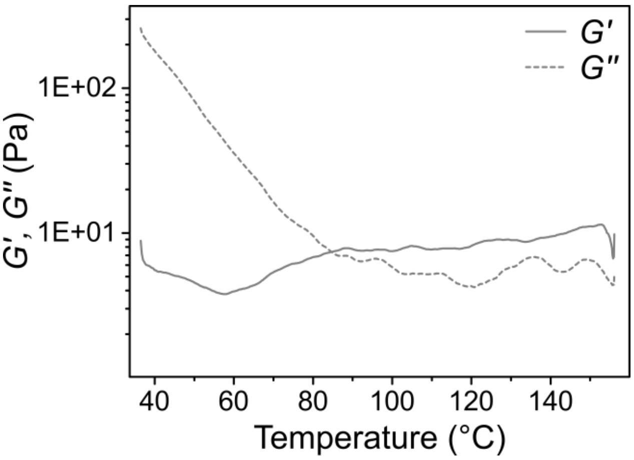

Supplementary Figure 23 | Mechanical properties of blank, linear polymer P. Thermo-rheological behavior of P after external deprotection of fpP. Storage (*G*') and loss moduli (*G*'') of **P** as a function of temperature during heating at 0.02 $K{\cdot}s^{-1}$ from 40 °C to 160 °C with *G*'' > *G*' in a temperature range of 36 °C to 85 °C until a *G*' / *G*'' cross-over. For further characterization of **P** see Supplementary Methods and Supplementary Fig. 1, 2, 5, 9, 14, 22.

Supplementary Table 1 | Overview over mesh sizes of unlocked polymer network **X@P$_{ON}$** at 21 °C and of locked polymer network **X@P$_{OFF}$** formed after UV-light illumination described by a second correlation length ξ_2 with added explanation. Mesh sizes are determined by SAXS measurements (see Supplementary Fig. 12, 13 for scattering curves and Supplementary Note 3 for detailed explanation).

	**X@P$_{ON}$ at 21 °C**	**X@P$_{OFF}$ at 21 °C**	**Δξ_2 (nm)**
**ξ_2 (nm)**	1.21 ± 0.07	1.61 ± 0.10	**→ 0.40 ± 0.10**
**Interpretation**	Higher probability of ring-closure at small mesh sizes which disappear → widening of mesh size		

Supplementary Table 2 | Overview over mesh sizes of unlocked polymer network **X@P$_{ON}$** at 21 °C and after heating to 120 °C for 30 min to form **X$_{ON}$ + P** described by a second correlation length ξ_2 with added explanation. Mesh sizes are determined by SAXS measurements (see Supplementary Fig. 12 for scattering curves and Supplementary Note 3 for detailed explanation).

	**X@P$_{ON}$ at 21 °C**	**X@P$_{ON}$ → X$_{ON}$ + P at 120 °C**	**Δξ_2 (nm)**
**ξ_2 (nm)**	1.21 ± 0.07	1.97 ± 0.20	**→ 0.76 ± 0.21**
**Interpretation**	Reduction of number of crosslinks upon heating → widening of mesh size		

Supplementary Table 3 | Overview over mesh sizes of locked polymer network **X@P$_{OFF}$** at 21 °C and at 120 °C for 30 min described by a second correlation length ξ_2 with added explanation. Mesh sizes are determined by SAXS measurements (see Supplementary Fig. 13 for scattering curves and Supplementary Note 3 for detailed explanation).

	**X@P$_{OFF}$ at 21 °C**	**X@P$_{OFF}$ at 120 °C**	**Δξ$_2$ (nm)**
**ξ_2 (nm)**	1.61 ± 0.10	1.91 ± 0.11	**→ 0.30 ± 0.10**
**Interpretation**	Successful locking of crosslinking points upon heating → only slight widening of mesh size		

Supplementary Table 4 | Overview over mesh sizes of locked polymer network **X@P$_{OFF}$** at 21 °C and of reestablished unlocked polymer network **X@P$_{ON}$** reformed after visible-light illumination described by a second correlation length ξ_2 with added explanation. Mesh sizes are determined by SAXS measurements (see Supplementary Fig. 13, 20 for scattering curves and Supplementary Note 3 for detailed explanation).

	**X@P$_{OFF}$ at 21 °C**	**reestablished X@P$_{ON}$ at 21 °C**	**Δξ$_2$ (nm)**
**ξ_2 (nm)**	1.61 ± 0.10	1.27 ± 0.11	**→ 0.34 ± 0.11**
**Interpretation**	Higher probability of ring-opening at small mesh sizes upon visible-light illumination with appearance of smaller meshes in mean mesh size → decrease of mesh size → successful restoration of mesh sizes of original **X@P$_{ON}$**		

Supplementary Table 5 | Overview over mesh sizes of reestablished unlocked polymer network **X@P$_{ON}$** at 21 °C and after heating to 120 °C for 30 min to form **X$_{ON}$ + P** described by a second correlation length ξ_2 with added explanation. Mesh sizes are determined by SAXS measurements (see Supplementary Fig. 20 for scattering curves and Supplementary Note 3 for detailed explanation).

	**X@P$_{ON}$ at 21 °C**	**X@P$_{ON}$ → X$_{ON}$ + P at 120 °C**	**Δξ$_2$ (nm)**
**ξ_2 (nm)**	1.27 ± 0.11	1.93 ± 0.12	**→ 0.66 ± 0.12**
**Interpretation**	Reduction of number of crosslinks upon heating → widening of mesh size		

Supplementary Note 1 | Quantification of the Diels-Alder crosslinking reaction in the solid state with help of UV/vis spectroscopic measurements

The efficiency of the crosslinking reaction (*i.e.* the ratio of unreacted to reacted furan-moieties) was estimated from the indicative bands in the solid state UV/vis spectra of their respective ring-closed forms at the photostationary state. For that purpose the UV/vis spectrum of $\mathbf{X@P_{OFF,partially}}$ (Supplementary Fig. 19b) was further investigated, as it includes the bands of both ring-closed isomers ($\mathbf{X_{OFF}}$ and $\mathbf{X@P_{OFF}}$) and is representative with regard to the conditions employed for the scratching tests.

Before any quantification by peak deconvolution can be performed, the wavelength-scale has to be transformed to the linear photon energy scale according to Planck's hypothesis. Multiple peak fitting (gray, red, yellow lines, Supplementary Fig. 3, performed by Gaussian Peak Fitting, OriginPro 9.1) then yields the veritable absorption maxima of $\mathbf{X_{OFF}}$ ($Abs_{\lambda max} = 0.024$) at $\lambda_{max} = 546$ nm; $E_{Ph,max} = 2.3$ eV (red line) and $\mathbf{X@P_{OFF}}$ ($Abs_{\lambda max} = 0.159$) at $\lambda_{max} = 437$ nm, $E_{Ph,max} = 2.8$ eV (yellow line).

To gather information over the molar concentrations of the photochromic units in the solid state via Lambert-Beer's Law from these absorbances, we assumed that the molar absorptivities of the chromophores in the poly(lauryl methacrylate) matrix are equal to those of small-molecule reference compounds in ethyl acetate as this solvent exhibits similar chemical properties as the bulk polymer.

For this purpose, molar absorptivities of $\mathbf{X_{OFF}}$ ($\varepsilon_{510\ nm} = 40400\ (M{\cdot}cm)^{-1}$) as well as of $\mathbf{Ref_{OFF}}$ ($\varepsilon_{428\ nm} = 9400\ (M{\cdot}cm)^{-1}$) in ethyl acetate after irradiation to the photostationary state with light of 365 nm of a LED were calculated hypothesizing 100% conversion to the ring-closed forms in the photostationary state, according to Göstl *et. al* [15].

With the assumption that the four photochromic units in $\mathbf{X@P_{OFF}}$ do not influence each other upon photoconversion, we tentatively state that one photochromic unit in $\mathbf{X@P_{OFF}}$ exhibits the same molar absorptivity as $\mathbf{Ref_{OFF}}$, i.e. $\varepsilon_{428\ nm,\mathbf{X@POFF}} = 4{\cdot}9400\ (M{\cdot}cm)^{-1} = 37600\ (M{\cdot}cm)^{-1}$.

We calculated the molar concentration of $\mathbf{X_{OFF}}$ $c_{\mathbf{XOFF},bulk}$ in the solid state via $Abs_{\lambda max} = \varepsilon_{510\ nm,\mathbf{XOFF}} \cdot d \cdot c_{\mathbf{XOFF},bulk}$ according to Lambert-Beer's Law, by employing a mean polymer film thickness of $d \approx 2.0$ µm which was determined by AFM measurements in the tapping mode. Hence, a molar concentration of $\mathbf{X_{OFF}}$ $c_{\mathbf{XOFF},bulk} \approx 2.97{\cdot}10^{-3}$ M was obtained. The same procedure was done for determination

of the molar concentration of **X@P$_{OFF}$** in the solid state via Lambert-Beer's Law $Abs_{\lambda\text{max}} = \varepsilon_{428\text{ nm},\mathbf{X@POFF}} \cdot d \cdot c_{\mathbf{XOFF},\text{bulk}}$ resulting in $c_{\mathbf{X@POFF},\text{bulk}} \approx 2.11 \cdot 10^{-2}$ M. With that ratio of molar concentrations of $c_{\mathbf{X@POFF}}$ and $c_{\mathbf{XOFF}}$, we assume approx. 88 % of reacted furans from **X@P$_{OFF}$** in the network **X@P$_{OFF,partially}$** in contrast to 12 % free furan moieties resulting from unreacted crosslinker.

Supplementary Note 2 | Solid state ^{1}H-NMR spectroscopy of unlocked polymer network X@P$_{ON}$

The procedure for polymer network preparation of **X@P$_{ON}$** was equal to typical scratching tests, followed by pestling the bulk material in liquid nitrogen media and storing for 16 h under low pressure atmosphere to obtain a powder of **X@P$_{ON}$**. Recording FT-IR spectra before and after milling shows no changes proving no structural destruction of the material. The ^{1}H MAS spectrum (Supplementary Fig. 4a) shows two strong signals at $\delta = 0.88$ ppm and $\delta = 1.28$ ppm belonging to the predominating protons of the poly(lauryl methacrylate) backbone (Supplementary Fig. 4b). Further zoom-in reveals signals at $\delta \approx 3.14$ ppm (**1** in Supplementary Fig. 4c) and $\delta \approx 6.66$ ppm (**2** in Supplementary Fig. 4c), which can be assigned to the Diels-Alder crosslinking motif. Thus, additional evidence for successful crosslinking in **X@P$_{ON}$** is provided. However, due to an overlapping and rather broad signals, a quantification to determine the amount of crosslinking efficiency, could not be carried out.

Supplementary Note 3 | Small-angle X-ray scattering measurements of X@P$_{ON}$ and X@P$_{OFF}$ in a complete light-controlled locking and de-locking cycle

Polymer networks produce characteristic SAXS (small-angle x-ray scattering) patterns which provide information on their structure according to the theory originally derived by de Gennes[16] and further developed by Panyukov and Rabin[17]. Hence, we assume that the scattering pattern differs among the different states of the polymeric network structure. Accordingly, changes of the polymeric mesh in network **X@P$_{ON}$**, **X@P$_{OFF}$** and reestablished **X@P$_{ON}$** at r.t. as well as after heating to the retro Diels-Alder reaction

temperature were determined via small angle x-ray scattering. Resultant scattering curves and appropriate mesh sizes are compared in Supplementary Fig. 5, 12, 13, 20, Supplementary Tables 1-5 as well as in this Supplementary Note.

Linear P and crosslinked X@$\mathbf{P_{ON}}$

First, the resultant scattering curves of blank linear polymer **P** without added crosslinker (gray solid line, Supplementary Fig. 5) and the curve of unlocked crosslinked polymer **X@**$\mathbf{P_{ON}}$ (black solid line) are compared at 21 °C to clarify all scattering contributions as well as the difference between non-crosslinked and crosslinked material at ambient temperature. We assign three characteristic regions in the scattering pattern of crosslinked **X@**$\mathbf{P_{ON}}$, labeled as 1, 2 and 3 (Supplementary Fig. 5), whereas in the scattering curve of linear polymer **P** only scattering contributions 1 and 3 occur.

Scattering contribution 1

Region 1 is dominated by a forward scattering, $I_1(q)$, with a sharp increase at q–values lower than 0.2 nm^{-1}. The cyan dashed line (Supplementary Fig. 5) represents the curve fit of scattering contribution $I_1(q)$ in **X@**$\mathbf{P_{ON}}$. $I_1(q)$ can be interpreted as resultant from large scale inhomogeneities of the bulk polymers and is present in crosslinked **X@**$\mathbf{P_{ON}}$ and linear **P**. Tentatively, it should be characterized by a first correlation length ξ_1.

Scattering contribution 2

The second scattering contribution, $I_2(q)$, can be interpreted as resultant from the network and is characterized by its entanglement distance. The contribution of $I_2(q)$ is clearly visible in the total scattering of the crosslinked polymer **X@**$\mathbf{P_{ON}}$ between 0.3 nm^{-1} < q < 1.2 nm^{-1}. The green solid line (Supplementary Fig. 5) represents the curve fit of scattering contribution $I_2(q)$ in **X@**$\mathbf{P_{ON}}$ which is by factor of approx. 3 larger than the scattering of non-crosslinked **P**. We interpret this as a characteristic of the network **X@**$\mathbf{P_{ON}}$ which is not present in linear **P**. Therefore, the mesh size of this network can be described by a second correlation length ξ_2.

Scattering contribution 3

Region 3 of the scattering pattern is dominated by a broad peak, $I_3(q)$, with a maximum at q_{max} around 2.5 nm^{-1} and is fitted by a magenta dash-dotted curve (Supplementary

Fig. 5). This broad peak is present in the scattering pattern of **X@P$_{ON}$** as well as in **P** and is therefore not a characteristic of the network. The presence of such a peak is well known for concentrated polymer systems that display microphase separation such as block copolymers in the amorphous state[18,19]. The underlying physical effect is referred to as "correlation-hole" effect. A reasonable interpretation for the presence of the same peak in network **X@P$_{ON}$** as well as in linear **P** is to assume that the dodecyl chains of the poly(lauryl methacrylate) backbone are microphase separated from the polymer backbone in these domains. The maximum diameter of these alkyl chain rich domains can be estimated[20] as two-times the alkyl chain lengths, which is $2l_c$ = (11×0.1265 + 0.15) nm = 3.08 nm. We quantified the broad peak by a characteristic distance of $d = 2\pi/q_{max}$ and a correlation length ξ_3.

*Physical background and discussion of results for **X@P$_{ON}$** and **P***

Taking all effects into account, we approximate the total scattering $I(q)$ as a sum of the three scattering contributions

$$I(q) = I_1(q) + I_2(q) + I_3(q). \quad (1)$$

In particular, the Debye-Büche function is used for $I_1(q)$, the Ornstein-Zernike function for the crosslinking contribution $I_2(q)$ and a Lorentzian peak function for $I_3(q)$, resulting in

$$I(q) = \frac{k_1}{[1+(\xi_1 q)^2]^2} + \frac{k_2}{1+(\xi_2 q)^2} + \frac{k_3}{[1+(\xi_3 |q-q_{max}|)^2]^2} \quad (2)$$

with scaling parameters k_1, k_2, and k_3 representing the maximum contribution to the respective scattering intensity, correlation lengths ξ_1, ξ_2, and ξ_3, and q_{max} as the peak position. The approach of utilizing the first two terms has been applied often, for example, by Matsunaga *et al.* for the interpretation of small-angle neutron scattering data of tetra-arm PEG gels[21]. We refer to their study and references therein[22] for a more detailed discussion of the scattering of polymeric gels as the most studied polymeric networks.

During the fitting procedure we found ambiguous values for ξ_1 because it is larger than our limit of determination. The maximum size available is given as $\xi_{1,max} = \pi/q_{min}$. From the smallest q-value of q_{min} = 0.06 nm^{-1} we determined $\xi_{1,max} = \pi/q_{min}$ of 52 nm. This value was used as fixed parameter for all curve fittings to avoid ambiguous results.

It should be noted that polymer networks often exhibit a hierarchical structure, with the largest dimensions on a length scale of greater than 100 nm, which is not accessible with SAXS. Here USAXS (ultra small-angle X-ray scattering) is a useful technique that allows information on structure hierarchy as described by Zhang and Ilavski[23]. But USAXS was not used in the present study and information on structure hierarchy is not available. Nevertheless, we found good fit results using $I(q)$ with the remaining parameters for the crosslinked polymer **X@P$_{ON}$** at 21 °C as shown in Supplementary Fig. 5 and already mentioned in this Supplementary Note. The second correlation length of **X@P$_{ON}$**, i.e. the correlation length of the mesh of **X@P$_{ON}$** has a mean value of ξ_2 = (1.21 ± 0.07) nm. The parameter values of the peak are ξ_3 = (0.76 ± 0.03) nm and q_{max} = (2.69 ± 0.02) nm^{-1}, which corresponds to a distance of d = 2.33 nm at 21 °C.

X@P$_{ON}$ at r.t. and at 120 °C

Comparison of the scattering curves of **X@P$_{ON}$** at 21 °C and after 30 minutes at 120 °C reveals a shift of scattering contribution 2 to lower q-values (blue and green filled areas in Supplementary Fig. 12), and thus, an increase of the correlation length from ξ_2 = (1.21 ± 0.07) nm at 21 °C to ξ_2 = (1.97 ± 0.20) nm at 120 °C.

We interpret this finding as a widening of the mesh size by $\Delta\xi_2$ = (0.76 ± 0.21) nm and conclude that at 120 °C the occurrence of a retro Diels-Alder reaction strongly reduces the number of crosslinks (**X@P$_{ON}$** $\rightarrow$ **X$_{ON}$** + **P**) (Supplementary Table 2). This is supported by the results of the scattering curves of crosslinked **X@P$_{ON}$** and linear **P** where scattering contribution 2 is not present in non-crosslinked **P**.

The broad peak also shifts to a higher value of q_{max} = (2.95 ± 0.02) nm^{-1}, which corresponds to d = 2.13 nm, and broadens which reflects in a lower correlation length of ξ_3 = (0.65 ± 0.02) nm. We interpret the slight difference of peak position and shape as a result of an increased thermal disorder in the lauryl-chain domains.

X@P$_{ON}$ and X@P$_{OFF}$ at r.t. with tentative explanation of mesh sizes

The mesh size ξ_2 = (1.21 ± 0.07) nm of non-illuminated **X@P$_{ON}$** at 21 °C increases to ξ_2 = (1.61 ± 0.10) nm after UV-light illumination due to the formation of locked **X@P$_{OFF}$** (for both scattering curves see black curves in Supplementary Fig. 12 and 13). This finding seems to be a contradiction at first sight. The UV-light induced ring-closure shortens the molecular length of the diarylethene units and should therefore

intuitively also reduce the correlation length ξ_2. Nevertheless, we tentatively assume that ring-closure has a higher probability at small meshes of the network due to a higher crosslinking and hence a higher photoswitch concentration (Supplementary Table 1). For this reason, the mean mesh size can increase due to an apparent disappearance of smaller meshes as they fall below the SAXS lower size detection limit of $d_{min} = \frac{\pi}{q_{max}} > 0.7 - 0.8\,nm$. The scattering intensity becomes too noisy at scattering vectors larger than $q_{max} = 4nm^{-1}$.

Furthermore, with SAXS, it is not possible to distinguish between a mesh resulting from chemically crosslinked polymer chains or from physical entanglements of chains or a mixture of both. Due to that, we must assume that a physical reorganization of the network structure is also included (for a detailed investigation of this nanostructural complexity see reference 24).

X@P$_{OFF}$ at r.t. and at 120 °C

The scattering curves of locked polymer network **X@P$_{OFF}$** at 21 °C and 120 °C reveal only a slight shift of scattering contribution 2 to lower *q*-values (yellow and red filled areas in Supplementary Fig. 13) and thus, a slight mesh widening from a correlation length of ξ_2 = (1.61 ± 0.10) nm at 21 °C to ξ_2 = (1.91 ± 0.11) nm after 30 minutes at 120 °C (Supplementary Table 3). In comparison to unlocked **X@P$_{ON}$** where the mesh widens with an amount of $\Delta\xi_2$ = (0.76 ± 0.21) nm the respective polymeric mesh in **X@P$_{OFF}$** hence only widens in a strongly reduced manner ($\Delta\xi_2$ = (0.30 ± 0.10) nm). We assume that due to a locking of crosslinking points in **X@P$_{OFF}$** after UV-light illumination, the polymeric mesh does not widen at these locked sites upon heating to 120 °C. The retro Diels-Alder reaction is inhibited.

X@P$_{OFF}$ and reestablished X@P$_{ON}$ at r.t.

Upon illumination with visible light, **X@P$_{OFF}$** is converted back to **X@P$_{ON}$** due to the ring-opening reaction of the diarylethene units. Herein, the correlation length decreases from ξ_2 = (1.61 ± 0.10) nm for **X@P$_{OFF}$** to ξ_2 = (1.27 ± 0.11) nm for reestablished **X@P$_{ON}$** (for both scattering curves see black curves in Supplementary Fig. 13 and 20). Thus, the original mesh size of non-illuminated **X@P$_{ON}$** (ξ_2 = (1.21 ± 0.07) nm) is

restored in reestablished **X@P_{ON}** confirming the reconstruction of the original chemical nature of the network (Supplementary Table 4).

Reestablished X@P_{ON} at r.t. and at 120 °C

Comparing the scattering curves of reestablished **X@P_{ON}** at 21 °C and after 30 minutes at 120 °C, a shift of scattering contribution 2 to lower *q*-values is revealed (blue and green filled areas in Supplementary Fig. 20). The mesh size of ξ_2 = (1.27 ± 0.11) nm of reestablished **X@P_{ON}** at 21 °C widens to ξ_2 = (1.93 ± 0.12) nm after 30 minutes at 120 °C (Supplementary Table 5). This difference of $\Delta\xi_2$ = (0.66 ± 0.12) nm) confirms a successful unlocking of crosslinking points after visible-light illumination resulting in a reestablished full widening of the mesh at higher temperatures in **X@P_{ON}** (compare original **X@P_{ON}** with $\Delta\xi_2$ = (0.76 ± 0.21) nm upon heating to 120 °C).

Supplementary Note 4 | Photoconversion of X@P_{OFF} in the photostationary state

UV/vis spectra of a small molecule reference compound, either in its 100% ring-open (**Ref_{ON}**, blue dashed line, Supplementary Fig. 11) or in its 100% ring-closed state (**Ref_{OFF}**, yellow dashed line) were recorded in degassed acetonitrile ($c \approx 10^{-5}$ M). For detailed structural information on **Ref_{ON}** and **Ref_{OFF}**, see Supplementary Fig. 16. The UV/vis spectrum of **X@P_{ON}** (blue solid line) is normalized to the peak maximum of **Ref_{ON}** at 330 nm (where the blank sample **P** absorbs insignificantly). The thus derived factor is used to normalize **X@P_{OFF}**, so that the maxima of the diagnostic bands of **X@P_{OFF}** and **Ref_{OFF}** can be compared with respect to the amount of formed ring-closed Diels-Alder adduct. This analysis indicates ca. 83% photoconversion of **X@P_{OFF}** in the photostationary state in thin films (Supplementary Fig. 11). Note that this value represents an upper limit of the amount of ring-closed isomer in the PSS and it should be lower in thicker films due to reduced optical penetration. Polymer film thickness for UV/vis spectroscopical measurements is approx. 2.0 µm, determined by AFM measurements in the tapping mode.

Supplementary Note 5 | DSC measurements of polymer networks X@P_{ON} and X@P_{OFF}

In DSC measurements, the de-crosslinking of the unlocked polymer network **X@P_{ON}** and the locked network **X@P_{OFF}** was recorded as an endothermic peak due to the removal of crosslinking points caused by the endothermic retro Diels-Alder reaction in a temperature range of 90 °C to 180 °C (Supplementary Fig. 7). The energy required for the endothermic de-crosslinking reaction, i.e. the reaction enthalpy ΔH_R, can be quantified by integrating the area of the respective peak[2,25].

In **X@P_{OFF}**, a number of crosslinking points are being locked by UV-light and hence the retro Diels-Alder reaction is inhibited at these sites. The respective endotherm is therefore lowered and the associated smaller integral of the corresponding peak in the DSC curves confirms the reduced reaction enthalpy. Assuming a more or less homogeneous distribution of crosslinking points, the relative amount of crosslinking points, which are locked upon UV-light illumination, can be estimated by the ratio of the measured reaction enthalpies.

In the first heating cycle, the integrated area in **X@P_{OFF}** is 33% lower (peak area = 82.9 mJ, ΔH_R = 9.3 J·g^{-1}) than in **X@P_{ON}** (peak area = 123.8 mJ, ΔH_R = 13.6 J·g^{-1}) (Supplementary Fig. 7). In the second heating cycle, a difference of 38% is calculated (for **X@P_{ON}**: peak area = 82.3 mJ, ΔH_R = 9.0 J·g^{-1}; while for **X@P_{OFF}**: peak area = 51.3 mJ, ΔH_R = 5.7 J·g^{-1}) confirming the result of the first heating cycle. Thus, it can be assumed, that around 1/3 of the crosslinking points are locked and cannot undergo the de-crosslinking reaction in the bulk material.

Supplementary Note 6 | Stability of X@P_{OFF} towards ambient light conditions investigated via UV/vis spectroscopy

UV/vis-spectra of sunlight with and without the filter window glass (Supplementary Fig. 18a) were measured with a portable USB2000+UV-vis-ES spectrometer from Ocean Optics coupled with a 400 µm optic fiber (for sunlight only, integration time: 1 ms) or without optic fiber (sunlight + filter window glass, integration time: 26 ms) to point out spectral differences in the UV-light region (Supplementary Fig. 18a). Data was recorded with the Ocean Optics SpectraSuite Software.

Supplementary Methods

All starting materials were used as supplied. Solvents were distilled prior to use. For work under inert conditions, HPLC grade solvents (Acros) were dried and degassed via a Pure Solv solvent purification system from Innovative Technologies. Dried and degassed glassware was flushed with argon several times. Work with diarylethene-type compounds was done under red light.

Liquid-NMR spectra were obtained on a 500 MHz (126 MHz for ^{13}C) Bruker AVANCE II 500 spectrometer or on a 300 MHz (75 MHz for ^{13}C) Bruker DPX 300 spectrometer at 25 °C.

Solid state NMR experiments were performed on a Bruker AVANCE 400 spectrometer using a 2.5 mm Bruker MAS probe. Spin-echo experiments were performed with a recycle delay (D_1) of 60 s and a scan number (ns) of 32. Calibration for ^{1}H-NMR spectroscopy was carried out with reference substances (adamantane as secondary standard with $\delta = 1.79$ ppm against TMS). The sample was rotated with $\nu_{rot} = 25$ kHz.

Ultrahigh-performance liquid chromatography / mass spectrometry (UPLC / MS) was performed on a Waters Acquity UPLC equipped with a Waters LCT Premier XE Mass for HR-MS (high-resolution MS) and with Waters Alliance systems consisting of a Waters Separations Module 2695, a Waters Mass Detector ZQ 2000 and a Waters Photodiode Array Detector 2996.

For **TLC-analysis** Merck Silica Gel 60 F254 TLC plates with a fluorescent indicator and with an excitation wavelength of 254 nm were used.

Column chromatography was performed with silica gel (35-70 µm, 60 Å, Acros) and with basic aluminium oxide (Brockmann I, 50-200 µm, 60 Å, Acros) for purification of polymers.

Size-exclusion chromatography (SEC) was either carried out on a WGE Dr. Bures system (UV detector: 230 nm, Knauer UV 2500, RI detector: Knauer RI K2301) equipped with three 300 x 8 mm SDV columns (50 Å 5 µm PSS, 500 Å 5 µm PSS, 1000 Å 5 µm PSS) and one 50 x 8 mm SDV column (eluent: THF, flow rate of 1 mL·min^{-1} at 60 °C) using several narrow dispersity–polystyrene standards or on a Shimadzu system (UV detector: SPD-10AD VP, RI detector: RID-10A) equipped with two PSS GRAM (1000/30 Å, 10 µm particle size) columns in series (eluent: DMAc with 2.1 g·L^{-1} LiCl; flow rate of 1 mL·min^{-1} at 40 °C) using linear PMMA standards. Received chromatograms were not corrected after the Mark-Houwink-equation.

General procedure for preparation of polymer films of X@P$_{ON}$ and X@P$_{OFF}$ for rheological, DSC and SAXS measurements

Copolymer **P** (1.0 equiv. maleimide side chains) and crosslinker **X$_{ON}$** (0.7 equiv. furan termini) were dissolved in a minimum amount of dry THF in a 2.5 mL vial and subsequently drop-casted onto a glass slide positioned in a Schlenk flask under an argon atmosphere followed by subsequent evacuation. The evacuated Schlenk flask was heated at 130 °C for 90 min to evaporate remaining solvent and to anneal the polymer mixture, followed by thermal crosslinking at r.t. for additional 16 h in the evacuated Schlenk flask.

For rheology, drop-casting was performed directly onto steel plates. Note that polymer network preparation has been optimized for thin films and no free-standing films (which would allow a classical tensile test) have been obtained.

After thermal crosslinking, half of the bulk material was irradiated with a Roithner 365 nm-LED XSL-365-5E at 20 mA and 4.2 V LED in a distance of 3 cm orthogonal to the sample and used as the locked polymer network sample **X@P$_{OFF}$** for further analysis. Furthermore, the locked and heated polymer film was irradiated with a LED Engin 460 nm Blue LED Emitter LZ4-00B208 at 2-3 mA and 12 V for 105 min to further investigate the reestablishment of the healability.

General procedure of scratching tests of X@P$_{ON}$ and X@P$_{OFF}$

Scratches were done with a scalpel in a controlled manner in the mm-scale, followed by masking half of the scratch and crosslinked polymer film **X@P$_{ON}$** (prepared as described above) with aluminum foil. Illumination of the unmasked area was performed

in a Schlenk flask under an argon atmosphere for 30 minutes by adjusting the above-mentioned Roithner 365 nm in a distance of 3 cm orthogonal to the glass slide to form **X@P$_{OFF}$**. After removal of the aluminium foil, the glass slide was heated on a Deben Enhanced Coolstage from ambient temperatures to 124 °C in a specimen chamber vacuum (about 30-50 Pa) of a SEM TM-1000 control unit remaining at that temperature for 5 min.

For reestablishment of the healability, the polymer film was irradiated with the above-mentioned LED Engin 460 nm-Blue LED Emitter for 105 min, followed by another heating process (same conditions as for locking the polymer).

Optical micrographs were acquired after every step.

Additionally, **FT-IR-spectra** were recorded after each step.

Thermogravimetric analysis (TGA) was carried out using a Netzsch TG 209 F1 in a temperature range from 20 °C to 600 °C.

Differential scanning calorimetry (DSC) was carried out either on a Perkin Elmer DSC 8500 Hyper-enabled Double-Furnace Differential Scanning Calorimeter in a temperature range from 0 °C to 180 °C or a DSC 204 F1 Phoenix by Netzsch from -100 °C to 180 °C, each with a heating rate of 20 $K \cdot min^{-1}$ under a nitrogen atmosphere. Baseline correction is performed by using linear interpolation in OriginPro 9.1, OriginLab Corp., Northampton, USA.

Optical micrographs were either acquired on a Bruker A670 Hyperion FT-IR microscope in the reflection mode with a visible objective, 40-times magnified (10x ocular, 4x vis-objective, for micrographs in main article) or with the optical detection unit of the AFM facility (for micrographs presented in the SI). The white balance of all images was automatically adjusted with a macro written for the Fiji software, provided by the light microscopy facility of the Cambridge Institute for Cancer Research, UK.

Fourier-transform infrared (FT-IR) spectroscopy was carried out on a Bruker Vertex 70v equipped with a Specac Golden Gate single reflection diamond ATR sample holder. Scans (number of scans: 128) were collected with a resolution of 4 cm^{-1} from 4000 to

400 cm^{-1}. Baseline correction was performed by using spline interpolation in OriginPro 9.1, OriginLab Corp., Northampton, USA.

Rheological measurements were performed on a Modular Advanced Rheometer System (HAAKE MARS II) of Thermo Fisher Scientific GmbH, Karlsruhe. An oscillatory shear mode with parallel plate geometry and 20 mm-diameter disposable aluminum plates was utilized for all experiments. A constant deformation of 0.1% shear strain at a frequency of 1 Hz was used. For Fig. 3e and Supplementary Fig. 14a, 23 experiments were done with a heating ramp rate of 0.02 $K{\cdot}s^{-1}$ from 40 °C to 160 °C by collecting data points every 10 s. A fixed value of 0.12 mm of sample thickness was set. For Supplementary Fig. 8 and 21 a constant normal force of 0.2 N was applied for gap control. Heating ramps were conducted at ca. 0.5 $K{\cdot}s^{-1}$ utilizing a Peltier element and collecting data points every 7 to 15 s with 3 repetitions per data point. Data analysis was performed with the software HAAKE RheoWin 4.3. Rheological data were smoothed using the Adjacent-Averaging Method in OriginPro 9.1, OriginLab Corp., Northampton, USA, except of graphs in Supplementary Fig. 8 and 21b. Note that compression or tensile measurements could not be performed due to the film's high viscoelasticity and creep even at low temperature.

SAXS measurements were performed in a solid sample holder with a Kratky-type instrument (SAXSess from Anton Paar, Austria) at temperatures of (21 $\pm$ 1) °C and (120 $\pm$ 2) °C. The SAXSess has a low sample-to-detector distance (0.309 m), which is appropriate for short measurement times of 10 min. The measured intensity was corrected by subtracting the intensity of the empty sample holder with a 30 µm thick aluminum foil. The scattering vector is defined in terms of the scattering angle θ and the wavelength of the radiation (λ = 0.154 nm): thus $q = 4\pi n/\lambda \sin\theta$. Deconvolution (slit length desmearing) of the SAXS curves was performed with the SAXS-Quant software. Curve fitting was conducted with the software *SASfit*[26].

For **solid state UV/vis-spectroscopy** of blank sample **P**, crosslinker $\mathbf{X_{ON}}$ **/** $\mathbf{X_{OFF}}$ in poly(LMA) and of polymer networks $\mathbf{X@P_{ON}}$ **/** $\mathbf{X@P_{OFF}}$ 3-5 µL of a solution of free crosslinker $\mathbf{X_{ON}}$ (0.6 and 0.7 equiv. furan per maleimide unit) (no crosslinker for blank sample preparation) and the respective polymer in degassed THF were spin-coated on

1 x 1 cm quartz glass plates (thickness of plates: 1 mm; polymer film thickness approx. 2.0 µm, determined by AFM measurements in tapping mode). Spin coating was performed at a rotation speed of 100-150 rps with a spin coating time set to 60 s with a KLM spin coater SCC-200 from SCHAEFER Technologies Corporation (Langen, Germany) at room temperature. Thermal crosslinking was carried out for 16 h at r.t. Irradiation was performed directly in a Varian Cary 50 UV/vis spectrophotometer equipped with a Peltier thermostated cell holder at 25 ± 0.05 °C by adjusting the LED in a distance of 1 cm orthogonal to the quartz glass plate in the sample holder. A Roithner 365 nm-LED XSL-365-5E for ring-closing at 20 mA and 4.2 V and a LED Engin 460 nm-Blue LED Emitter LZ4-00B208 for ring-opening at 2-3 mA and 12 V were employed, both driven by a GW Instek GPD-3303S linear DC power supply.

Irradiation experiments of the small molecule reference compound **Ref$_{\mathbf{ON}}$** in degassed Acetonitrile of spectroscopic grade, ($c \approx 10^{-5}$ M) were done on a preparative scale on a Rayonet RPR 100 photochemical reactor equipped with 300 nm lamps. The ring-closing reaction to yield **Ref$_{\mathbf{OFF}}$** was monitored by UV/vis spectroscopy on a Varian Cary 50 UV/vis spectrophometer equipped with a Peltier thermostated cell holder at 25 ± 0.05 °C in a 3 mL quartz cuvette.
Further purification to yield 100% ring-closed isomer **Ref$_{\mathbf{OFF}}$** was performed via preparative high-performance liquid chromatography.

Synthesis of crosslinker X_{ON}

The tetrafuryl-substituted DAE-type crosslinker $\mathbf{X_{ON}}$ was synthesized via a Suzuki cross coupling reaction of the precursor DAE[15] with **I**:

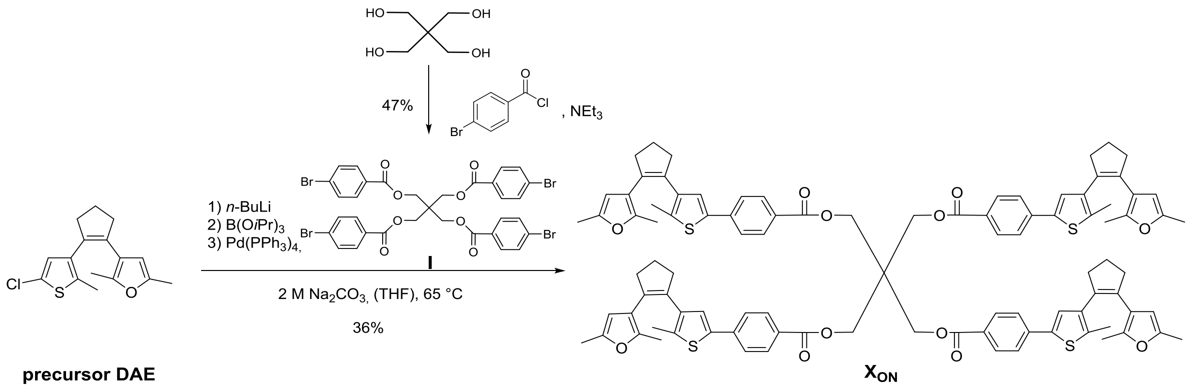

A five-step synthesis including a Michael-type addition yields the **precursor DAE** (1-(3-(2,5-dimethylfuryl))-2-(3-(5-chloro-2-methylthienyl)-cyclopentene, whose synthesis was recently described by our research group[15]. Suzuki cross coupling reaction of the **precursor DAE** and tetra(4-bromobenzoylmethyl)methane **I**, which was synthesized by four-fold esterification of pentaerythritol with 4-bromobenzoyl chloride, provided the tetrafunctional DAE crosslinker in its ring-open form ($\mathbf{X_{ON}}$).

Tetra(4-bromobenzoylmethyl)methane (**I**)

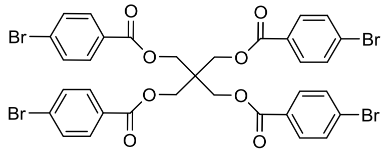

To an ice-cooled solution of pentaerythritol (0.23 g, 1.70 mmol, 1 equiv.) and dry triethylamine (1.13 mL, 8.16 mmol, 4.8 equiv.) in 3 mL of dry dichloromethane was added a freshly prepared solution of 4-bromobenzoyl chloride (1.79 g, 8.16 mmol, 4.8 equiv.) in 3 mL of dichloromethane dropwise via an addition funnel under an argon atmosphere. The resulting mixture was stirred at r.t. for 21 h. Afterwards, the reaction mixture was diluted with sat. aq. Na_2CO_3 solution. The organic layer was washed with brine and dried over $MgSO_4$, and the solvent was removed under reduced pressure. The crude mixture was dissolved in dichloromethane and filtrated over a pad of silica. The

filtrate was concentrated in vacuum to yield the target compound as a white solid (47%).

^{1}H-NMR (300 MHz, $CDCl_3$): δ 7.84-7.79 (m, 8H, $4x2xCH_{ar}$), 7.56-7.52 (m, 8H, $4x2xCH_{ar}$), 4.65 (s, 8H, $4xCH_{2\text{-symm}}$); ^{13}C-NMR (75 MHz, $CDCl_3$): δ 165.4, 132.1, 131.2, 128.9, 128.2, 63.7.

Tetrafunctional photoswitchable crosslinker in reactive ring-open form ($\mathbf{X_{ON}}$)

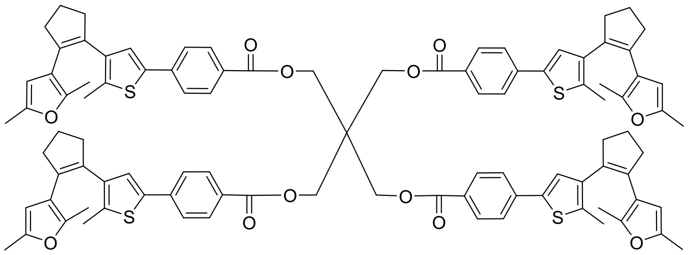

A solution of *n*-BuLi (1.28 mL, 2.81 mmol, 2.2 M in hexane, 1.1 equiv.) was added dropwise to a solution of the **precursor DAE** (747 mg, 2.55 mmol, 1 equiv.) in 4 mL of dry THF in a dry Schlenk flask at r.t. under an argon atmosphere. The reaction mixture turned dark red and was stirred at r.t. for 30 min. Afterwards, triisopropyl borate (0.88 mL, 3.83 mmol, 1.5 equiv.) was added and the mixture was stirred for another 1.5 h. Meanwhile, tetra(4-bromobenzoylmethyl)methane **I** (251 mg, 0.59 mmol, 0.23 equiv.) was dissolved in 4 mL of THF in a 10 mL dry Schlenk flask and tetrakis(triphenylphosphine)palladium(0) (295 mg, 0.26 mmol, 0.1 equiv.) was added in one portion under argon flow. The resulting yellow mixture was stirred at r.t. for 1 h. Then, 5.2 mL of a degassed 2 M aq. Na_2CO_3 solution and 1 drop of ethylene glycol were added. The resulting two-phase system was heated to reflux. The resulting borate was added to this solution via a syringe without any work-up. Subsequently, the combined solutions were stirred at 65 °C for 20 h. Afterwards, the reaction mixture was quenched with water, extracted three times with dichloromethane and dried over $MgSO_4$. The solvent was removed under reduced pressure. The crude product was purified by column chromatography (petroleum ether : dichloromethane = 1 : 3) to yield the desired crosslinker $\mathbf{X_{ON}}$ as a white solid (36%).

^{1}H-NMR (500 MHz, toluene-d_8): δ 8.02 (d, J = 8.4 Hz, 8H, $4x2xCH_{ar}$), 7.31 (d, J = 8.4 Hz, 8H, $4x2xCH_{ar}$), 7.12 (s, 4H, $4xCH_{thio}$), 5.74 (s, 4H, $4xCH_{fur}$), 4.70 (s, 8H,

4xCH$_{2_symm}$), 2.70 (m, 16H, 4x2xCH$_2$), 2.05 (s, 12H, 4xC$_{ar}$CH$_3$), 1.96 (d, 24H, 2x4xC$_{ar}$CH$_3$), 1.95 – 1.89 (m, 8H, 4xCH$_2$); ^{13}C-NMR (126 MHz, toluene-d$_8$): δ 166.0, 150.0, 147.4, 139.7, 139.6, 138.6, 136.4, 133.0, 132.6, 131.2, 129.6, 128.7, 128.6, 126.5, 125.6, 119.0, 107.3, 64.4, 44.0, 39.5, 38.4, 23.6, 14.9, 13.7, 13.6; HRMS (m/z): [M-H]$^-$ calcd. for [$C_{97}H_{92}O_{12}S_4$], 1575.5393; found 1575.6412.

Synthesis of monomer MIMA and blank, linear polymer P

Atom-transfer radical polymerization (ATRP)[27,28] of a furyl-protected maleimide methacrylate (**fpMIMA**) and lauryl methacrylate (LMA) yields the furan-protected random copolymer poly(LMA-*co*-fpMIMA) **fpP**, which can be deprotected to the reactive, un-masked poly(LMA-*co*-MIMA) **P** by heating at 130 °C for 3 h.

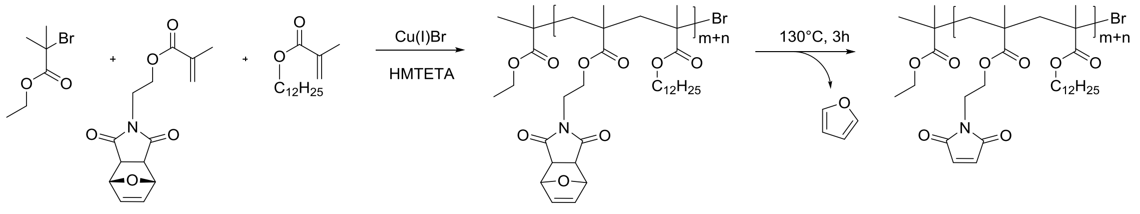

EB*i*B **MIMA** **LMA** **fpP** **P**

The employed masked maleimide methacrylate (**fpMIMA**) monomer was prepared in three synthetic steps by adapted reported procedures[27,29,30].

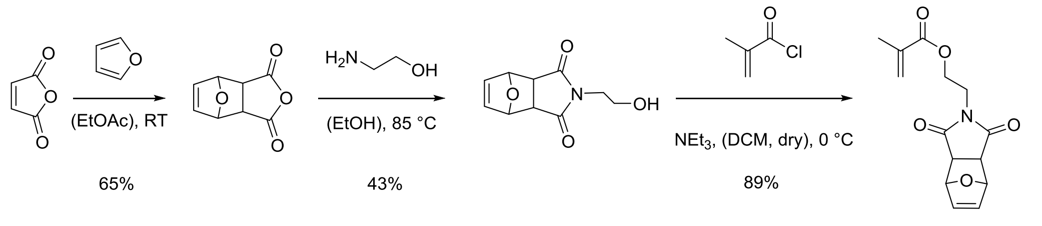

fpMIMA

3a,4,7,7a-Tetrahydro-4,7-epoxyisobenzofuran-1,3-dione

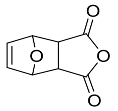

Furan (21.82 mL, 300.0 mmol, 1 equiv.) was added to a solution of maleic anhydride (22.09 g, 225.3 mmol, 0.75 equiv.) in 13 mL ethyl acetate and the reaction mixture was stirred for 24 h at r.t. The formed white precipitate was filtered off and dried in vacuum to yield mainly the *exo*-compound as a white solid (65%).

^{1}H-NMR (300 MHz, DMSO-d_6): δ 6.59 (t, J = 0.9 Hz, 2H, CH=CH), 5.36 (t, J = 0.9 Hz, 2H, CH-O-CH), 3.32 (s, 2H, CH-CH).); ^{13}C-NMR (75 MHz, DMSO-d_6): δ 171.6, 136.9, 81.7, 49.1.

2-(2-Hydroxyethyl)-3a,4,7,7a-tetrahydro-1H-4,7-epoxyisoindole-1,3(2*H*)-dione

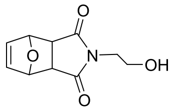

To a suspension of 4,10-dioxatricyclo[5.2.1.0$^{2.6}$]dec-8-ene-3.5-dione (32.32 g, 194.6 mmol, 1 equiv.) in 58 mL of ethanol, 2-aminoethanol (12.24 mL, 202.4 mmol, 1.04 equiv.) was added dropwise leading to formation of a clear solution. The reaction mixture was heated to 85 °C for 4.5 h and was then allowed to cool to r.t. and stirred for another 12 h. The formed white precipitate was filtered off to obtain mainly the *exo*-compound as a white solid (43%).

^{1}H-NMR (300 MHz, $CDCl_3$): δ 6.51 (t, J = 0.9 Hz, 2H, CH=CH), 5.27 (t, J = 0.9 Hz, 2H, CH-O-CH), 3.78-3.71 (m, 2H, CH_2OH), 3.71-3.63 (m, 2H, NCH_2), 2.88 (s, 2H, CH-CH), 2.42 (br, s, 1H, -OH); ^{13}C-NMR (75 MHz, $CDCl_3$): δ 176.7, 136.4, 80.9, 60.1, 47.4, 41.6.

2-(1,3-Dioxo-3a,4,7,7a-tetrahydro-1H-4,7-epoxyisoindol-2(3H)-yl)ethyl methacrylate (**fpMIMA**)

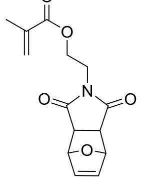

In a 25 mL Schlenk flask under an argon atmosphere, methacryloyl chloride (0.15 mL, 1.5 mmol, 1.05 equiv.) was added dropwise to a solution of 2-(2-hydroxyethyl)-3a,4,7,7a-tetrahydro-1H-4,7-epoxyisoindole-1,3(2*H*)-dione (0.31 g, 1.5 mmol, 1 equiv.) and triethylamine (0.25 mL, 1.8 mmol, 1.2 equiv.) in 7 mL of dry dichloromethane at 0 °C. The reaction mixture was stirred for 2 h at 0 °C. The reaction mixture was diluted and extracted with dichloromethane and washed two times with an aq. $NaHCO_3$ solution and water. After removal of the solvent under reduced pressure, a colorless waxy oil was obtained. A short silica column (ethyl acetate : dichloromethane = 4 : 5) provided a white solid, which was identified as the *exo*-compound of **fpMIMA** (89%). 4-Methoxyphenol (30 ppm) was added as radical inhibitor before removing the solvent under reduced pressure.

^{1}H-NMR (300 MHz, $CDCl_3$): δ 6.51 (t, J = 0.9 Hz, 2H, CH=CH), 6.07 (dd, J = 1.6, 1.0 Hz, 1H, $C=CH_2$), 5.56 (p, J = 1.6 Hz, 1H, $C=CH_2$), 5.26 (t, J = 0.9 Hz, 2H, CH-O-CH), 4.32-4.24 (m, 2H, OCH_2), 3.85-3.77 (m, 2H, NCH_2), 2.86 (s, 2H, O=C-CH-CH-C=O), 1.90 (dd, $^3J_{H,H}$ = 1.6, 1.0 Hz, 3H, CH_3); ^{13}C-NMR (75 MHz, $CDCl_3$): δ 175.9, 166.9, 136.5, 135.8, 126.0, 80.8, 60.8, 47.4, 37.6 18.1.

Poly(LMA-*co*-fpMIMA) (**fpP**)

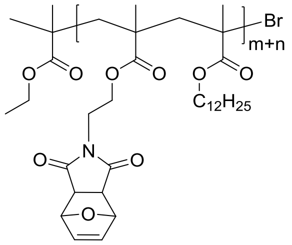

The reaction was carried out under inert conditions. Dry toluene was degassed by bubbling argon through the solution for 30 min. Greenish Cu(I)Br was purified several times with acetic acid and subsequently with methanol and then dried *in vacuo* for at least 12 h. The inhibitor in fpMIMA was removed by filtration over aluminum oxide prior to use.

Masked maleimide methacrylate (fpMIMA) (0.11 g, 0.40 mmol, 0.16 equiv.) and Cu(I)Br (16 mg, 110 µmol, 0.04 equiv.) were placed in a pressure tube equipped with a magnetic stir bar and kept under vacuum for 1 h. In a dry 25 mL Schlenk flask, lauryl methacrylate (0.74 mL, 2.51 mmol, 1 equiv.), HMTETA (30 µL, 110 µmol, 0.04 equiv.)

were dissolved in 2 mL of toluene. Argon was bubbled through the solution for several minutes. Afterwards, the reaction mixture was added to the solids in the pressure tube. After stirring at r.t. for 20 min, the resulting mixture was heated to 70 °C in an oil bath. The polymerization was started by adding ethyl α-bromoisobutyrate (EB*i*B) (16 µL, 110 µmol, 0.04 equiv.). The reaction mixture was stirred for 3 h at 70 °C whereas the solution turned greenish. The reaction was stopped subjecting the flask to air and by adding THF (2 mL). The catalyst and ligand were removed by passing through a basic aluminium oxide column, followed by a removal of the solvent under reduced pressure. To remove traces of remaining monomer the residue was dissolved in a minimum amount of toluene and precipitated in ice-cold methanol several times to afford poly(LMA-*co*-fpMIMA) (**fpP**) as a colorless viscous oil.

^{1}H-NMR (300 MHz, $CDCl_3$): δ 6.54 (s, br, -CH=CH-, furyl moiety), 5.28 (s, br, CH-O-CH), 3.90 (s, br, O-CH_2, LMA side chain), 3.75 (s, br, O-CH_2, N-CH_2, MIMA), 2.98 (s, br, O=C-CH-CH-C=O), 1.89 (s, br, CH, polymer backbone, MIMA), 1.79 (s, br, CH, polymer backbone, LMA), 1.61 (s, br, O-CH_2-CH_2-, LMA side chain), 1.27 (s, br, $(CH_2)_9$, LMA side chain), 1.12 (s, br, CH_3, polymer backbone, MIMA), 1.02 (s, br, CH_3, polymer backbone, LMA), 0.90-0.86 (t, br, CH_3, LMA side chain); ^{13}C-NMR (126 MHz, $CDCl_3$): δ 179.7, 177.6, 176.8, 138.1, 80.6, 64.8, 61.4, 54.0, 47.3, 44.8, 37.7, 31.7, 29.4, 29.3, 29.1, 27.9, 25.8, 22.4, 14.0.

SEC (THF, 40 °C) of the prepared polymers gave a number average molecular weight $\overline{M}_n$ between 6000 and 8600 g·mol^{-1} and a dispersity *Đ* between 1.17 and 1.26. The amount of masked maleimide methacrylate **fpMIMA** was determined via integration and comparison of diagnostic peaks in the NMR spectra and amounts to 10-12 mol%.

Poly(LMA-*co*-MIMA) (**P**)

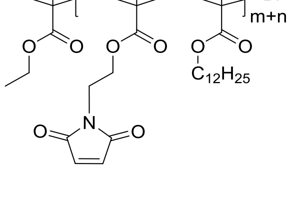

A bulk film of poly(LMA-*co*-fpMIMA) **fpP** was heated on a glass substrate in a drying oven at 130 °C for 3 h. ^{1}H NMR analysis showed quantitative conversion of the

oxabicyclic moiety to the maleimide functional group to obtain demasked poly(LMA-*co*-MIMA) **P**.

^{1}H-NMR (300 MHz, $CDCl_3$): δ 6.80 (s, br, CH=CH, MIMA side-chain), 3.91 (s, br, O-CH_2, LMA side-chain), 3.82 (s, br, O-CH_2, N-CH_2, MIMA), 1.91 (s, br, CH, polymer backbone, MIMA), 1.80 (s, br, CH, polymer backbone, LMA), 1.61 (s, br, O-CH_2-CH_2-, LMA side-chain), 1.27 (s, br, $(CH_2)_9$, LMA side-chain), 1.13 (s, br, CH_3, polymer backbone, MIMA), 1.03 (s, br, CH_3, polymer backbone, LMA), 0.90-0.86 (t, br, CH_3, LMA side-chain); ^{13}C-NMR (75 MHz, $CDCl_3$): δ 178.1, 177.4, 170.3, 134.3, 65.0, 61.4, 48.1, 43.4, 38.8, 32.2, 29.9, 29.8, 29.6, 28.4, 26.2, 23.0, 14.3.

Besides NMR measurements, the deprotection from **fpP** to **P** was monitored via FT-IR spectroscopy (Supplementary Fig. 22) as well as thermogravimetric analysis (TGA) and differential scanning calorimetry (DSC) both shown in Supplementary Fig. 1.

Supplementary References

1. Parker, S. F. Vibrational spectroscopy of N-phenylmaleimide. *Spectrochim. Acta. A. Mol. Biomol. Spectrosc.* **63,** 544–9 (2006).

2. Gaina, C., Ursache, O. & Varganici, C. D. Thermally reversible cross-linked poly(ether-urethane)s. *Express Polym. Lett.* **7,** 636–650 (2013).

3. Imai, Y., Itoh, H., Naka, K. & Chujo, Y. Thermally Reversible IPN Organic−Inorganic Polymer Hybrids Utilizing the Diels−Alder Reaction. *Macromolecules* **33,** 4343–4346 (2000).

4. Winter, H. H. Can the Gel Point of a Cross-linking Polymer Be Detected by the G' - G" Crossover? *Polym. Eng. Sci.* **27,** 1698–1702 (1987).

5. Adzima, B. J., Aguirre, H. A., Kloxin, C. J., Scott, T. F. & Bowman, C. N. Rheological and chemical analysis of reverse gelation in a covalently cross-linked diels-alder polymer network. *Macromolecules* **41,** 9112–9117 (2008).

6. Froidevaux, V. *et al.* Study of the Diels–Alder and retro-Diels–Alder reaction between furan derivatives and maleimide for the creation of new materials. *RSC Adv.* **5,** 37742–37754 (2015).

7. Sahu, U. S. & Bhadani, S. Homopolymerization of Maleimide. *Makromolecular Chemie Rapid Commun.* **3,** 103–107 (1982).

8. Bose, R. K. *et al.* A rheological and spectroscopic study on the kinetics of self-healing in a single-component diels-alder copolymer and its underlying chemical reaction. *J. Polym. Sci. Part A Polym. Chem.* **52,** 1669–1675 (2014).

9. Stevens, M. P. & Jenkins, A. D. Crosslinking of Polystyrene via Pendant Maleimide Groups. *J. Polym. Sci.* **17,** 3675–3685 (1979).

10. Aguiar, E. C., da Silva, J. B. P. & Ramos, M. N. A theoretical study of the vibrational spectrum of maleimide. *J. Mol. Struct.* **993,** 431–434 (2011).

11. Raghavan, S. R. *et al.* Rheological study of crosslinking and gelation in chlorobutyl elastomer systems. *Polymer (Guildf).* **37,** 5869–5875 (1996).

12. Parker, S. F. Vibrational spectroscopy of N-phenylmaleimide. *Spectrochim. Acta. A. Mol. Biomol. Spectrosc.* **63,** 544–549 (2006).

13. Thompson, H. & Temple, R. The infra-red spectra of furan and thiophen. *Trans. Faraday Soc.* **41,** 27–34 (1945).

14. Grigg, R., Knight, J. A. & Sargent, M. V. Studies in Furan Chemistry. Part I. The Infrared Spectra of 2,5-Disubstituted Furans. *J. Chem. Soc.* 6057–6060 (1965).

15. Göstl, R. & Hecht, S. Controlling covalent connection and disconnection with light. *Angew. Chem. Int. Ed. Engl.* **53,** 8784–8787 (2014).

16. De Gennes, P.-G. *Scaling Concepts in Polymer Physics*. (Cornell University Press: Ithaca and London, 1979).

17. Panyukov, S. & Rabin, Y. Statistical physics of polymer gels. *Phys. Rep.* **269,** 1–131 (1996).

18. Leibler, L. Theory of microphase separation in block copolymers. *Macromolecules* **13,** 1602–1617 (1980).

19. Sweat, D. P. *et al.* Phase Behavior of Poly(4-hydroxystyrene- block -styrene) Synthesized by Living Anionic Polymerization of an Acetal Protected Monomer. *Macromolecules* **47,** 6302–6310 (2014).

20. Oliver, R. C. *et al.* Dependence of Micelle Size and Shape on Detergent Alkyl Chain Length and Head Group. *PLoS One* **8,** e62488 (2013).

21. Matsunaga, T., Sakai, T., Akagi, Y., Chung, U. Il & Shibayama, M. SANS and SLS studies on tetra-arm PEG gels in as-prepared and swollen states. *Macromolecules* **42,** 6245–6252 (2009).

22. Hammouda, B., Ho, D. & Kline, S. SANS from Poly(ethylene oxide)/Water Systems. *Macromolecules* **35,** 8578–8585 (2002).

23. Zhang, F. & Ilvasky, J. Ultra-Small-Angle X-ray Scattering of Polymers. *Polym. Rev.* **50,** 59–90 (2010).

24. Seiffert, S. Effect and evolution of nanostructural complexity in sensitive polymer gels. *Macromol. Chem. Phys.* **216,** 9–22 (2015).

25. Toncelli, C., De Reus, D. C., Picchioni, F. & Broekhuis, A. A. Properties of Reversible Diels-Alder Furan/Maleimide Polymer Networks as Function of Crosslink Density. *Macromol. Chem. Phys.* **213,** 157–165 (2012).

26. Breßler, I., Kohlbrecher, J. & Thünemann, A. F. *SASfit*: a tool for small-angle scattering data analysis using a library of analytical expressions. *J. Appl. Crystallogr.* **48,** 1587–1598 (2015).

27. Syrett, J. A., Mantovani, G., Barton, W. R. S., Price, D. & Haddleton, D. M. Self-healing polymers prepared via living radical polymerisation. *Polym. Chem.* **1,** 102–106 (2010).

28. Kavitha, A. A. & Singha, N. K. Atom-Transfer Radical Copolymerization of Furfuryl Methacrylate (FMA) and Methyl Methacrylate (MMA): A Thermally-Amendable Copolymer. *Macromol. Chem. Phys.* **208,** 2569–2577 (2007).

29. Heath, W. H. *et al.* Degradable Cross-Linkers and Strippable Imaging Materials for Step-and-Flash Imprint Lithography. *Macromolecules* **41,** 719–726 (2008).

30. Dispinar, T., Sanyal, R. & Sanyal, A. A Diels-Alder / Retro Diels-Alder Strategy to Synthesize Polymers Bearing Maleimide Side Chains. *J. Polym. Sci. Part A Polym. Chem.* **45,** 4545–4551 (2007).
